# Tributyltin Protumorigenic Effects Targeting Prostate Cancer Cell Metabolism, Proliferation, Migration, and Invasion

**DOI:** 10.1002/tox.70029

**Published:** 2026-01-06

**Authors:** Mariana Feijó, Luís P. Brás, Catarina M. D. Serra, Lara R. S. Fonseca, Sara Correia, Bruno J. Pereira, Ana P. Duarte, Henrique J. Cardoso, Cátia V. Vaz, Sílvia Socorro

**Affiliations:** ^1^ RISE‐Health, Department of Chemistry, Faculty of Sciences University of Beira Interior Covilhã Portugal; ^2^ RISE‐Health, Department of Medical Sciences, Faculty of Health Sciences University of Beira Interior Covilhã Portugal; ^3^ Faculty of Health Sciences University of Beira Interior Covilhã Portugal; ^4^ Instituto Português de Oncologia de Coimbra Francisco Gentil Coimbra Portugal

**Keywords:** 5α‐dihydrotestosterone, endocrine‐disrupting‐chemicals, low‐density lipoprotein, prostate cancer, tributyltin

## Abstract

Prostate cancer (PCa) is an endocrine‐related cancer highly dependent on androgenic signaling. Beyond hormone dependence, extrinsic factors play a significant role in the risk of developing PCa, which raises concern about the influence of environmental compounds such as endocrine‐disrupting chemicals (EDCs). Tributyltin (TBT) is an EDC used in antifouling paints, and its androgenic (and obesogenic) actions have been described. This study investigated the effect of TBT on various cancer hallmarks, specifically its impact on the viability, metabolism, proliferation, migration, and invasion of prostate cells, using in vitro and in vivo models. Androgen‐sensitive (LNCaP) and androgen‐insensitive (PC3) PCa cells were exposed to 1–100 nM TBT for 24 and 48 h. Additionally, LNCaP cells were treated with 100 nM TBT in the presence of the androgen receptor antagonist bicalutamide (1–40 μM) or 10 nM TBT and/or low‐density lipoprotein (LDL, 100 μg/mL) and 5α‐dihydrotestosterone (DHT, 10 nM, 48 h). Wistar rats were administered TBT (50 μg/kg) every 3 days for 45 days. TBT disrupted glycolytic flux and lipid handling in prostate cells, enhancing their proliferative activity. Moreover, 100 nM TBT stimulated migration and invasion of LNCaP cells. Bicalutamide attenuated the effect of TBT in inducing glucose consumption and LNCaP cell proliferation. A 10× lower TBT concentration maintained the stimulatory effects on LNCaP cells' viability, proliferation, and migration/invasion, sustained by high LDL‐cholesterol availability and DHT. Our results show TBT as a potential inducer of PCa progression and aggressiveness and contribute to increasing awareness about the roles of EDCs in the prostate carcinogenic process.

## 1 Introduction

Prostate cancer (PCa) is the second most common cancer in men and represents the fifth leading cause of cancer‐related mortality [1]. As an endocrine‐related cancer and in the first stages of the disease, PCa is highly dependent on androgenic signaling mediated by the androgen receptor (AR). The androgens/AR actions sustain cell survival and stimulate the proliferative activity of PCa cells, contributing to tumor growth [2, 3].

Beyond hormone dependence, the contribution of extrinsic factors to the risk of developing PCa has been estimated to be over 70% [4], which strongly raises concern about the influence of environmental compounds, such as endocrine‐disrupting chemicals (EDCs). EDCs are natural or synthetic compounds found in the environment and everyday‐use products capable of interfering with the normal function of the endocrine system, leading to adverse health effects [5]. Exposure to EDCs has been shown to elevate the risk of cancer, though their contribution to prostate malignancy is not clear [6]. EDCs are also disruptors of cell metabolism, being linked to the emergence of metabolic disorders [7]. The metabolic reprogramming and the plasticity of cancer cells to alter metabolism dependently on the environmental pressure is a pivotal hallmark of cancer [8]. Therefore, the threat of EDCs as protumorigenic agents becomes a “double trouble” as, besides affecting endocrine function, some of these compounds can disrupt metabolic homeostasis, namely, by favoring lipid handling toward fat accumulation, as is the case of the so‐called obesogens [9, 10]. The situation is even more worrying, considering EDCs produce impactful effects not only at acute or high doses [11, 12]. Experts highlight the threat of ubiquitous and chronic exposure to EDCs, with low exposure levels still having significant and long‐term biological impact, warning that there may be no “safe dose” [13].

The triorganotin derivative tributyltin (TBT) is an EDC used for decades in antifouling paint formulations because of its biocidal properties [14]. Although TBT usage was banned globally in 2008 [14], its pollution in coastal areas worldwide persists today, with several hotspots identified [15]. Therefore, exposure to TBT continues to be a current public health problem and a matter of concern.

TBT's actions have been described as obesogenic [16, 17, 18, 19] and potentially androgenic [20, 21, 22]. As an obesogen, TBT can dysregulate lipid metabolism, promoting adipogenesis [9, 16, 23], which can greatly impact tumor growth and invasiveness, as lipids are crucial structural components of the cell membrane, participate as second messengers in intracellular signaling and represent an important energy source [24]. A previous report also showed that exposure to TBT increased androgen‐dependent transcription and proliferation of androgen‐sensitive PCa cells [20], but its action over other biological features remains unclear. Based on its obesogenic and androgenic activity and PCa dependency on hormone actions, we hypothesize that TBT's tumorigenic potential and its mechanisms of action may target other cancer hallmarks. The present study investigated the effect of TBT on prostate cells and how it influences the various cancer hallmarks, such as cell viability, metabolism, cell proliferation, migration, and invasion. The analysis was complemented by determining the impact of lower TBT levels, depending on lipid availability and the presence of androgens.

## 2 Materials and Methods

### 2.1 Cell Lines and Treatments

The neoplastic human prostate cell lines, LNCaP and PC3, were obtained from the European Collection of Cell Cultures (ECACC, Salisbury, UK). LNCaP cells are derived from lymph node metastasis of PCa, express the AR, and are androgen‐sensitive [25]. PC3 cells have origin from bone metastasis and are considered to be nonsensitive to androgens [26], mimicking the stage of castration‐resistant PCa (CRPC). LNCaP and PC3 cell lines have been widely used as in vitro models of early androgen‐sensitive and late castration‐resistant PCa stages, respectively [27, 28].

LNCaP and PC3 cells were maintained in RPMI‐1640 phenol red culture medium (R6504, Sigma‐Aldrich, St. Louis, MO, USA), supplemented with 1% penicillin–streptomycin‐amphotericin B (A5955, Sigma‐Aldrich) and 10% fetal bovine serum (FBS, F7524, Sigma‐Aldrich) at 37°C in a humidified atmosphere of 5% (v/v) CO_2_. At 60% confluence, the culture medium was replaced by phenol red‐free RPMI 1640 medium (R8755, Sigma‐Aldrich) containing 5% charcoal‐stripped FBS (F6765, Sigma‐Aldrich) or 1% lipid‐depleted FBS (S148L, Biowest, Riverside, MO, USA) as it will follow treatment with TBT (T50202, CAS no: 1461‐22‐9, 96%, Sigma‐Aldrich)‐only (LNCaP and PC3 cells) or TBT + low‐density lipoprotein (LDL, 437644, CAS no: 308068‐14‐6, ≥ 95%, Merck, Darmstadt, Germany) + 5α‐dihydrotestosterone (DHT, D‐073, CAS no: 521‐18‐6, 97.9%, Sigma‐Aldrich) (LNCaP cells), respectively. Cells were maintained for an additional 24 h and then exposed to a TBT concentration range (0, 1, 10, and 100 nM) for 24 or 48 h. These selected TBT concentrations mimic the context of real situations, following the range found in human blood after chronic exposure, that is, 10–100 nM [29, 30]. The androgen‐sensitive LNCaP cells were also exposed to 100 nM TBT in the presence or absence of the AR antagonist bicalutamide (1, 10, 20, and 40 μM).

Another experimental approach included treatment of LNCaP with a low TBT concentration (10 nM) and/or LDL (100 μg/mL) and DHT (10 nM) for 48 h.

A TBT 10 mM stock solution was prepared in ethanol, and all TBT working solutions were obtained through serial dilutions from the stock solution in cell culture medium.

### 2.2 In Vivo Experiments

Adult Wistar ( *Rattus norvegicus* ) male rats (350–400 g; approximately 4 months old) were housed at constant temperature and humidity under a 12‐h light/dark cycle with food and water available *ad libitum*. The research was conducted according to the guidelines established by the Guide for the Care and Use of Laboratory Animals published by the U.S. National Institutes of Health (NIH Publication No. 85‐23, revised 1996), Guidance on the operation of the Animals (Scientific Procedures, Act 1986 ASPA), and the E.U. rules for the care and handling of laboratory animals (Decree‐Law No. 113/2013; E.U. Directive 2010/63). All animal experiments complied with ARRIVE guidelines. In addition, the animal protocol was approved by the local “FCS‐UBI Animal Welfare and Ethics Body” (ORBEA, *Orgão de Bem‐Estar e Ética Animal*) and authorized by the general directorate of food and veterinary (DGAV, *Direção‐Geral de Alimentação e Veterinária*). Rats were divided into two groups (*n* = 7), receiving TBT (50 μg/kg, T50202, CAS no: 1461‐22‐9, Sigma‐Aldrich) or vehicle (corn oil, C8267, Sigma‐Aldrich) administered by oral gavage every 3 days for 45 days. The TBT dose was defined according to previous studies, where TBT exposure resulted in increased body weight, visceral fat accumulation, and dyslipidaemia [19, 31, 32]. After treatment, animals were weighed and euthanized under anesthesia. Whole prostates were removed, weighed, and either fixed in 4% paraformaldehyde (PFA) or snap‐frozen in liquid nitrogen and stored at −80°C.

### 2.3 Cell Viability Assay

LNCaP (15 000 cells/well) and PC3 (4000 cells/well) cells were grown in 96‐well plates, and cell viability was assessed by the 3‐(4,5‐Dimethylthiazolyl‐2)‐2,5‐diphenyltetrazolium bromide (MTT) assay. After treatments, culture medium was removed and cells were incubated with MTT (0.5 mg/mL) in the dark for 4 h at 37°C. After incubation, the MTT solution was removed, and the formed formazan crystals were solubilized with 100 μL of dimethyl sulfoxide (DMSO). The absorbance of the resultant purple‐colored solution was measured at 570 nm using the xMark Microplate Absorbance Spectrophotometer (Bio‐Rad, Hercules, CA, USA). The value of absorbance is directly proportional to the number of viable cells.

### 2.4 Sulforhodamine B (SRB) Assay

LNCaP (25 000 cells/well) cells were grown in 96‐well plates, and cell mass was assessed by the SRB assay. SRB (S1402, Sigma‐Aldrich) is a bright pink aminoxanthene dye that binds to basic amino acids of cell proteins, functioning as an indicator of proliferation. After TBT and/or bicalutamide treatment, the cell culture medium was removed, and cells were fixed in 12% cold trichloroacetic acid (TCA). The TCA solution was removed, and cells were washed with distilled water and dried at room temperature. 100 μL of SRB (0.05%) was added to each well, and the plate was incubated in the dark for 1 h at 37°C. Unbound dye was removed with 1% acetic acid. The dye bound was solubilized with 200 μL of 10 mM Tris base solution at pH 10. The plate was shaken for 10 min, and the absorbance was measured at 540 nm using the xMark Microplate Absorbance Spectrophotometer (Bio‐Rad). The value of absorbance obtained is directly proportional to cell mass.

### 2.5 Real‐Time Polymerase Chain Reaction (qPCR)

Total RNA from control and TBT‐treated LNCaP was isolated using the TripleXtractor reagent (GRiSP, Oporto, Portugal) according to the manufacturer's instructions. The quantity and integrity of total RNA were determined by measurement of absorbance at 260 and 280 nm (Nanodrop One, Thermo Fisher Scientific, Rockford, USA) and agarose gel electrophoresis. cDNA was synthesized from 1 μg total RNA using the Reliance Select cDNA Synthesis kit in a 20 μL reaction volume (12012801, Bio‐Rad), following the manufacturer's instructions. qPCR was used to assess the expression of the prostate‐specific antigen (PSA)‐encoding gene kallikrein‐related peptidase 3 (*KLK3*) using the CFX Connect Real‐Time PCR Detection System (Bio‐Rad). *β‐2‐microglobulin* (*β2M*) was the housekeeping gene used to normalize gene expression, following the Pfaffl mathematical model and using the 2^−(ΔΔCt)^ formula. The details regarding primers, cycling conditions, and annealing temperature are presented in Table 1.

**TABLE 1:** Primers and real‐time polymerase chain reaction (qPCR) cycling conditions.

Gene (GenBank accession number)	Primer sequence (5′–3′)	Primer concentration (nM)	Amplicon size (bp)	AT (°C)	Amplification cycles (*n*)
Kallikrein‐related peptidase 3 (NM_001030047.4)	Sense: AGGCCTTCCCTGTACACCAA Antisense: GTCTTGGCCTGGTCATTTCC	300	133	60	40
*β‐2‐microglobulin* (NM_004048.2)	Sense: ATGAGTATGCCTGCCGTGTG Antisense: CAAACCTCCATGATGCTGCTTAC	300	93	60	40

Abbreviation: AT, annealing temperature.

### 2.6 Quantification of Glucose and Lactate

The concentration of glucose and lactate in LNCaP and PC3 cell culture medium was assessed by spectrophotometric analysis using commercial assay kits (41010, 1001330, Spinreact, Girona, Spain) according to the manufacturer's instructions. 1 μL of cell culture medium collected at 0 and 48 h after the addition of TBT and/or bicalutamide (LNCaP) and 100 μL of the respective kit work reagent were mixed in a well plate and incubated at 37°C for 10 and 5 min for glucose and lactate quantification, respectively. The absorbance values were measured at 505 nm using the xMark Microplate Absorbance Spectrophotometer (Bio‐Rad).

Glucose consumption and lactate production were determined by comparing the metabolite content in the culture medium at 0 and 48 h and normalizing to the total amount of cell protein in each experimental group.

### 2.7 Total Protein Extraction

Prostate cells (LNCaP and PC3) and tissue were homogenized in an appropriate volume of radioimmunoprecipitation assay buffer (RIPA, 150 mM NaCl, 1% Nonidet‐P40 substitute, 0.5% Na‐deoxycholate, 0.1% SDS, 50 mM Tris, 1 mM EDTA), supplemented with 1% protease inhibitors cocktail (A7779, PanReac AppliChem, Darmstadt, Germany) and 10% PhosSTOP (4906845001, Roche, Mannheim, Germany), kept on ice for 20 min with occasional mixing and then centrifuged at 18800 *g* for 20 min at 4°C. The supernatant containing total proteins was collected, and protein concentration was quantified using the Bicinchoninic acid (BCA) Protein Assay Kit (23 225, Thermo Fisher Scientific), according to the manufacturer's instructions.

### 2.8 Western Blot (WB) Analysis

Total proteins (25 μg) were denatured at 100°C for 5 min and resolved by sodium dodecyl sulfate‐polyacrylamide gel electrophoresis (SDS–PAGE) on 10% polyacrylamide gels. Proteins were electro‐transferred to polyvinylidene difluoride (PVDF) membranes (Bio‐Rad) at 4°C (750 mA) for 2 h. Membranes were blocked with 5% skimmed dried milk for 1 h at room temperature and incubated overnight at 4°C with rabbit anti‐glucose transporter 2 (GLUT2, 1:500, H‐67, sc‐9117, Santa Cruz Biotechnology, Heidelberg, Germany), rabbit anti‐phosphofructokinase‐1 (PFK1, 1:1000, H‐55, sc‐67 028, Santa Cruz Biotechnology), rabbit anti‐lactate dehydrogenase (LDH, 1:10000, ab52488, EP1566Y, Abcam, Cambridge, UK), rabbit anti‐monocarboxylate transporter 4 (MCT4, 1:1000, H‐90, sc‐50 329, Santa Cruz Biotechnology), rabbit anti‐CD36 (1:400, ab64014, Abcam), rabbit anti‐acetyl‐CoA carboxylase (ACC, 1:1000, #3662S, Cell Signaling Technology, Danvers, Massachusetts, EUA), rabbit anti‐fatty acid synthase (FASN, 1:1000, C20G5, #3180S, Cell Signaling Technology), mouse anti‐carnitine‐palmitoyl transferase‐1A (CPT1A, 1:1000, 8F6AE9, ab 128 568, Abcam), rabbit anti‐Akt (1:500, #9272; Cell Signaling Technology), rabbit anti‐Phospho‐Akt (1:500, #9271; Cell Signaling Technology), rabbit anti‐MAPK (1:500, #9102; Cell Signaling Technology), rabbit anti‐Phospho‐MAPK (1:500, #9101; Cell Signaling Technology), rabbit anti‐Phospho‐C‐Myc (1:500, #13748; Cell Signaling Technology), rabbit anti‐AR (1:250, sc‐816, Santa Cruz Biotechnology), or mouse anti‐E‐cadherin (1:1000, sc‐8426, Santa Cruz Biotechnology) primary antibodies. Blots were sequentially incubated with a maximum of two primary antibodies whenever the protein molecular weight allowed for proper resolution in target separation. A mouse anti‐β‐actin monoclonal antibody (1:10000, A1978, Sigma‐Aldrich) was used for protein loading control. Membranes were then incubated for 1 h with the goat anti‐rabbit IgG HRP‐linked (1:10000, #7074S, Cell Signaling Technology) or m‐IgGk BP‐HRP (1:20000, sc‐516 102, Santa Cruz Biotechnology) secondary antibodies. Subsequently, membranes were incubated with ECL substrate (Bio‐Rad) for 5 min and scanned with the Chemidoc MP Imaging System (Bio‐Rad). Band densities were obtained and quantified by the volumetric analysis tool of Image Lab 5.1 software (Bio‐Rad) and normalized by division with the respective β‐actin band density.

### 2.9 LDH Activity Assay

The enzymatic activity of LDH was determined using a commercial assay kit (41222, Spinreact) according to the manufacturer's instructions. The capacity that the LDH present in the sample has to catalyze the reduction of pyruvate into lactate with the oxidation of NADH to NAD^+^ was determined by measuring the rate of decrease in NADH levels. Briefly, protein extracts were added to a 96‐well plate with a previously prepared kit reagent in a 1:150 proportion and incubated at 37°C for 1 min. Absorbance at 340 nm was measured using the xMark Microplate Absorbance Spectrophotometer (Bio‐Rad) at the initial time‐point, and then consecutive absorbance readings were acquired at 1‐min intervals for 3 min. LDH activity is directly proportional to the variation of absorbance over 3 min in each sample. The activities achieved were calculated as U/L/μg protein.

### 2.10 Oil Red‐O Assay

The Oil Red‐O method was used to detect and quantify intracellular neutral lipid droplets in prostate cells [33]. LNCaP (200 000 cells/well) and PC3 (50 000 cells/well) cells were fixed with 4% PFA for 30 min, washed twice with distilled water and rinsed in 60% isopropanol for 5 min. After washing, cells were stained with Oil Red‐O solution (O1391, Sigma‐Aldrich) for 15 min. Representative images were acquired on the Olympus CKX41 inverted phase contrast microscope under 20× magnification. For quantification of lipid content, Oil Red‐O was eluted from cells with 100% isopropanol under gentle agitation for 5 min, and absorbance was measured at 492 nm using the xMark Microplate Absorbance Spectrophotometer (Bio‐Rad).

### 2.11 Ki‐67 Fluorescent Immunocytochemistry

LNCaP cells (150 000/well) were fixed with 4% PFA and permeabilized with 1% Triton X‐100 for 5 min at room temperature. Unspecific staining was blocked by incubation with PBS containing 0.1% (w/v) Tween‐20 (PBS‐T) and 20% FBS for 1 h. After washing, cells were incubated with the rabbit anti‐Ki‐67 (1:50, ab16667, Abcam) primary antibody for 1 h at room temperature. Alexa Fluor 546 goat anti‐rabbit IgG (1:1000, Invitrogen, Carlsbad, CA, USA) was used as the secondary antibody (1 h at room temperature). The specificity of staining was assessed by the omission of the primary antibody. Cell nuclei were stained with Hoechst 33342 (5 μg/mL, H3570, Invitrogen) for 10 min. After additional washing with PBS‐T, lamellae were mounted in Dako fluorescent mounting medium (Dako, Glostrup, Denmark). Images were acquired using the Zeiss LSM 710 laser scanning confocal microscope (Carl Zeiss, Göttingen, Germany). The proliferation index was determined by the percentage of Ki‐67‐positive cells out of the total number of Hoechst‐stained nuclei in 10 randomly selected 400× magnification fields in each lamella.

### 2.12 Ki‐67 Fluorescent Immunohistochemistry

Formalin‐fixed paraffin 5 μm sections of rat prostate were deparaffinized in xylene and rehydrated through a series of graded ethanol solutions. After heat‐induced antigen retrieval in 10 mM citrate buffer (pH 6), sections were permeabilized with 1% Triton X‐100 for 15 min at room temperature. Unspecific staining was blocked by incubation with PBS containing 1% (w/v) BSA and 0.3 M glycine for 30 min at room temperature. Sections were incubated overnight at 4°C with rabbit anti‐Ki67 primary antibody (1:50, ab16667, Abcam). Alexa Fluor 546 goat anti‐rabbit IgG was used as a secondary antibody (1:500, Invitrogen, 1 h at room temperature). The specificity of the staining was assessed by the omission of the primary antibody. Cell nuclei were stained with Hoechst 33342 (10 μg/mL, H3570, Invitrogen) for 5 min. Sections were washed with PBS and mounted in Dako fluorescent mounting medium (Dako, Glostrup, Denmark). Images were acquired using a Zeiss LSM 710 laser scanning confocal microscope (Carl Zeiss). The proliferation index was determined by the percentage of Ki67‐positive cells out of the total number of Hoechst‐stained nuclei in 10 randomly selected 400× magnification fields in each section.

### 2.13 Migration and Invasion Assays

LNCaP cells (300 000/transwell) were plated on the upper chamber of Matrigel‐coated (354480, BD Biosciences, New Jersey, USA) and non‐coated chambers (35224, SPL, Life Sciences, Naechon‐Myeon Pocheon, South Korea) for migration and invasion analysis, respectively. Cells were cultured in serum‐free media with or without the respective treatments. The lower chambers contained media with 10% FBS as a chemoattractant. After exposure to TBT, cells on the lower surface of the transwell were fixed, washed, and stained with Hoechst 33342 (5 μg/mL, H3570, Invitrogen). Migration and invasion were assessed by the total number of Hoechst‐stained nuclei in 5 randomly selected 400× magnification fields per transwell.

### 2.14 Statistical Analysis

Statistical significance of differences between controls and TBT‐treated groups was evaluated by unpaired *T*‐test or one‐way ANOVA followed by Tukey post hoc test using GraphPad Prism v6.00 (GraphPad Software, San Diego, CA, USA). Differences were considered statistically significant when *p* < 0.05. All experimental data are shown as mean ± standard error of the mean (S.E.M.).

## 3 Results

### 3.1 TBT Exposure Increased the Viability of PCa Cells

**FIGURE 1:**
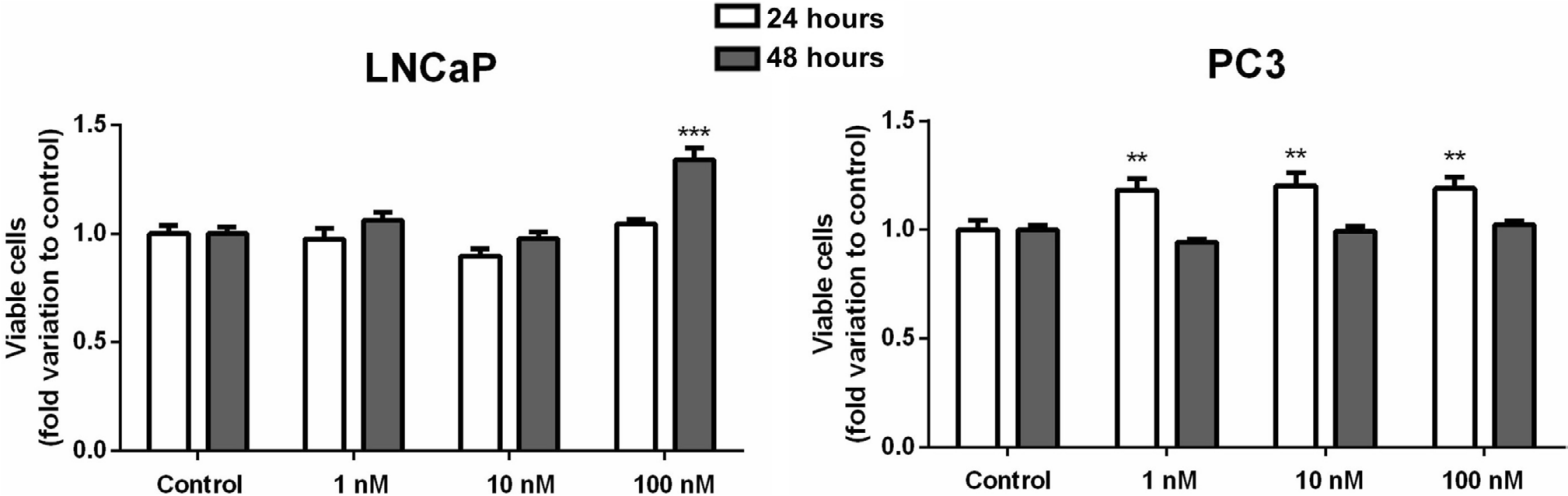
Effect of TBT (1, 10, or 100 nM) on the viability of androgen‐sensitive (LNCaP) and androgen‐insensitive (PC3) human neoplastic prostate cells. The percentage of viable cells after 24 and 48 h of treatment was evaluated by the MTT assay and was normalized relative to control. Results are expressed as a % of control. Error bars indicate mean ± S.E.M. (*n* ≥ 6). *p < 0.05; **p < 0.01; ***p < 0.001 compared with the control group.

The viability of neoplastic human prostate cells in response to TBT treatment was investigated by the MTT assay. In the androgen‐sensitive LNCaP cells, 100 nM TBT treatment for 48 h increased cell viability by approximately 34% (1.34 ± 0.05‐fold variation to the control group, Figure 1). Also, the viability of the CRPC PC3 cells was significantly increased after treatment with 1, 10, and 100 nM TBT for 24 h (1.18 ± 0.05‐, 1.20 ± 0.06‐, and 1.19 ± 0.05‐fold variation to the control, respectively, Figure 1).

### 3.2 TBT Enhanced the Glycolytic Profile of PCa Cells

The capacity of tumor cells to reprogram metabolism is a critical hallmark of cancer implicated in cell survival, tumor growth, and metastasis [8, 34]. We and others have previously reported that the sex steroid androgens can disrupt PCa cells' metabolism, enhancing their glycolytic profile and lipid metabolism [27, 28, 35, 36, 37, 38]. As mentioned, TBT is an EDC with androgen actions, which prompted us to investigate its effects on prostate cell metabolism.

**FIGURE 2:**
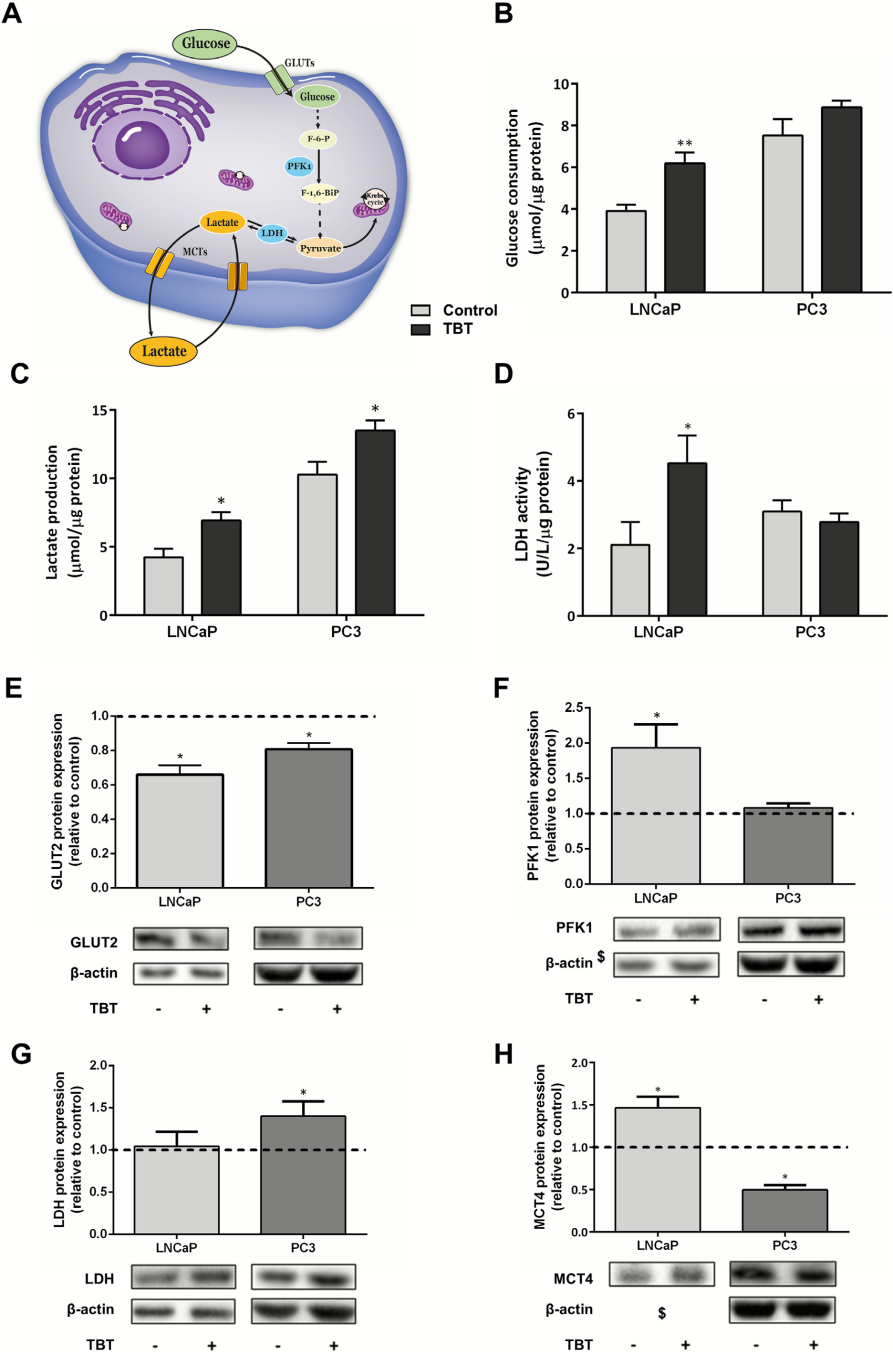
Effect of TBT in modulating glycolytic metabolism in androgen‐sensitive (LNCaP) and androgen‐insensitive (PC3) human neoplastic prostate cells. Cells were treated with 100 nM TBT for 48 h. (A) General overview of the glycolytic pathway. Glucose enters the cell through the activity of glucose transporters (GLUTs), being converted into pyruvate through the sequential activity of several enzymes, namely phosphofructokinase 1 (PFK1), which catalyze the conversion of fructose‐6‐phosphate (F‐6‐P) to fructose‐1,6‐bisphosphate (F‐1,6‐BiP). Pyruvate can enter the Krebs cycle in mitochondria or alternatively be metabolized into lactate by the activity of lactate dehydrogenase (LDH), a preferable route in highly glycolytic PCa cells. Lactate is exported to the extracellular space through monocarboxylate transporters (MCTs), namely MCT4. It can also be reused by cancer cells as a substrate. (B) Glucose consumption, (C) lactate production, and (D) LDH enzymatic activity determined by spectrophotometric assays. (E) GLUT2, (F) PFK1, (G) LDH, and (H) MCT4 expression determined by WB analysis. Results are expressed as fold variation relative to the control group (dashed line) after normalization with β‐actin (hexaplicates). $ PFK1 and MCT4 were sequentially labeled on the same blot, with protein levels being quantified by normalization with the same β‐actin bands. Representative immunoblots are shown as bottom panels. Error bars indicate mean ± S.E.M. (*n* = 6). *p < 0.05; **p < 0.01 compared with the control group.

Cell glycolytic flux (Figure 2A) is maintained by the glucose uptake from the extracellular space by GLUTs family members and its conversion to pyruvate through the sequential action of several enzymes, such as PFK1, which catalyzes a rate‐limiting step in glycolysis converting fructose‐6‐phosphate to fructose‐1,6‐bisphosphate [28, 39]. Highly glycolytic cancer cells, which in the case of PCa correspond to the advanced stages of the disease [27], preferentially channel pyruvate to be converted into lactate by the activity of LDH, instead of its mitochondrial oxidation (Figure 2A) [40, 41]. Although less efficient in ATP production, the conversion of pyruvate to lactate is faster in producing energy [42], and the produced lactate is exported to the extracellular space by the MCTs (Figure 2A).

Treatment with 100 nM TBT for 48 h enhanced glucose consumption in LNCaP cells (6.18 ± 0.53 vs. 3.91 ± 0.30 μmol/μg protein in the control group, Figure 2B), whereas no changes were observed in PC3 (Figure 2B). Regarding lactate production, a significant increase was observed in LNCaP and PC3 cells (6.93 ± 0.61 vs. 4.24 ± 0.63 μmol/μg protein in the control group and 13.50 ± 0.73 vs. 10.30 ± 0.92 μmol/μg protein in the control group, respectively, Figure 2C) after TBT treatment.

The activity of LDH, the enzyme that converts pyruvate into lactate (Figure 2A), was significantly increased in TBT‐treated LNCaP cells (4.53 ± 0.82 vs. 2.11 ± 0.68 U/L/μg protein in the control group, Figure 2D), with no alterations being perceived in PC3 cells.

The expression of key targets of glycolytic metabolism, GLUT2, PFK1, LDH, and MCT4 (Figure 2A) was evaluated by WB analysis. GLUT2 expression was significantly decreased in response to TBT treatment in the studied cell lines (0.66 ± 0.05‐ and 0.81 ± 0.04‐fold variation to the control group, respectively, Figure 2E). Relatively to PFK1, its expression increased significantly in TBT‐treated LNCaP cells with approximately 1.94‐fold variation to the TBT‐untreated group (Figure 2F). PFK1 expression levels remained unchanged in PC3 cells exposed to TBT (Figure 2F).

TBT significantly increased LDH expression in PC3 cells (1.40 ± 0.18‐fold variation to the control, Figure 2G). No significant alterations were found in the expression of LDH in LNCaP cells. The expression of MCT4 (Figure 2A) was significantly increased in LNCaP cells upon treatment with TBT (1.47 ± 0.13‐fold variation to the control group, Figure 2H). Contrarily, a significant decrease in MCT4 expression was verified in TBT‐treated PC3 cells (0.50 ± 0.06‐fold variation to the control group, Figure 2H).

### 3.3 The Anti‐Androgen Bicalutamide Attenuated the TBT‐Induced Effect in Stimulating Glucose Consumption in Androgen‐Sensitive PCa Cells

**FIGURE 3:**
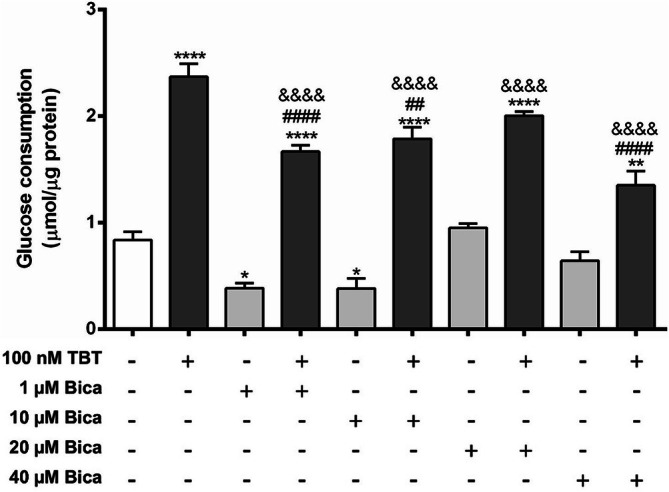
Bicalutamide (Bica) modulation of the TBT effects over glucose consumption in LNCaP cells. Cells were treated with 100 nM TBT in the presence or absence of a concentration range (1–40 μM) of Bica for 48 h. Error bars indicate mean ± S.E.M. (*n* = 6). *p < 0.05, **p < 0.01, ****p < 0.0001, when compared with the control group; ^##^ *p* < 0.01, ^####^ *p* < 0.0001, when compared with the TBT‐treated group; ^&&&&^ *p* < 0.0001, when compared with the respective Bica group (same concentration).

To infer the role of androgen signaling in the actions of TBT altering glucose handling, LNCaP cells were treated with 100 nM TBT for 48 h in the presence or absence of a concentration range (1, 10, 20, and 40 μM) of the AR inhibitor bicalutamide, and glucose consumption (Figure 3) was evaluated.

The presence of bicalutamide (except 20 μM) decreased the effect of TBT in increasing the glucose consumption of LNCaP cells (1.67 ± 0.06, 1.79 ± 0.11 and 1.35 ± 0.13 vs. 2.37 ± 0.12 μmol/μg protein in the TBT‐only group, respectively, for 1, 10, and 40 μM, Figure 3). Interestingly, bicalutamide alone (1 and 10 μM) also suppressed glucose consumption compared to the control group (0.39 ± 0.06 and 0.38 ± 0.10 vs. 0.84 ± 0.08 μmol/μg protein, respectively, Figure 3).

### 3.4 TBT Exposure Disrupted Lipid Handling in PCa Cells

**FIGURE 4:**
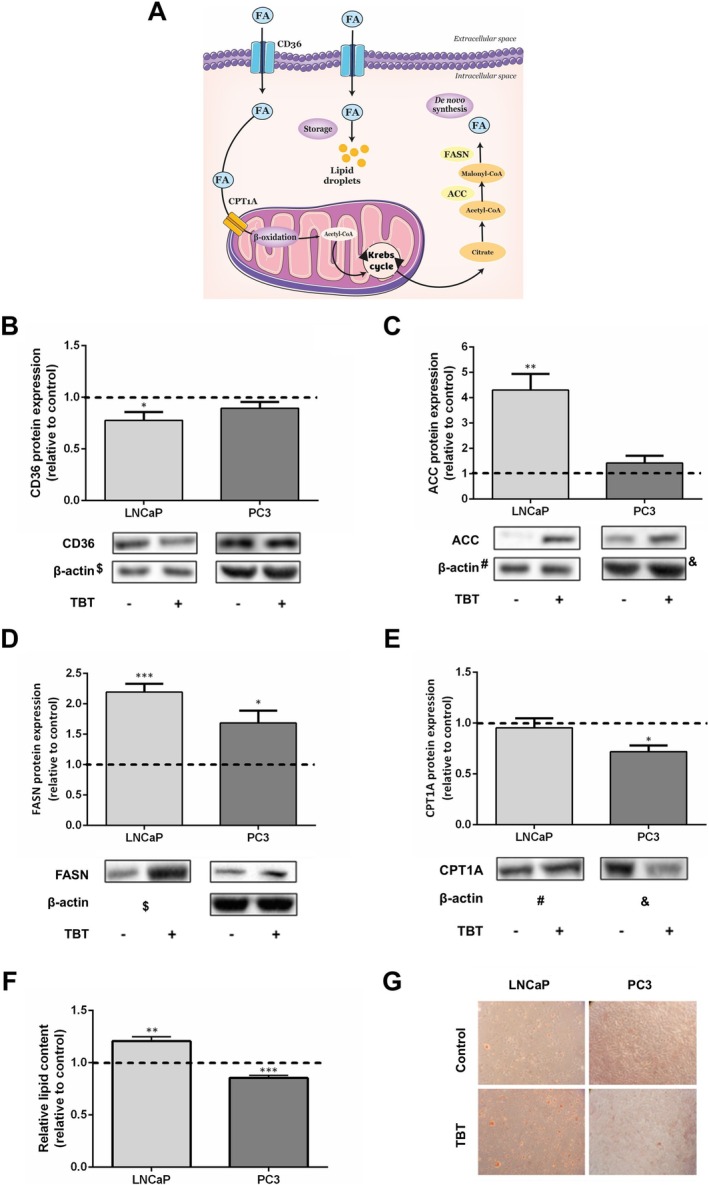
Effect of TBT in modulating lipid handling in androgen‐sensitive (LNCaP) and androgen‐insensitive (PC3) human neoplastic prostate cells. Cells were treated with 100 nM TBT for 48 h. (A) Lipid metabolism. Fatty acids (FA) uptake from the extracellular space by the CD36 transporter are stored in lipid droplets or undergo β‐oxidation. FA conjugated with carnitine by the activity of carnitine palmitoyltransferase 1 (CPT1A), are transported to the mitochondria generating acetyl‐CoA that enters the Krebs cycle. In PCa cells, de novo FA synthesis is an active metabolic pathway known to be dependent on citrate and the activity of acetyl‐CoA carboxylase (ACC) and FA synthase (FASN). (B) CD36, (C) ACC, (D) FASN, and (E) CPT1A protein expression determined by WB analysis after normalization with β‐actin (hexaplicates). CD36 and FASN ($), as well as ACC and CPT1A (# and &) were sequentially labeled on the same blot with protein levels being quantified by normalization with the same β‐actin bands. Representative immunoblots are shown as bottom panels. (F) Lipid content determined by the Oil Red‐O assay. (G) Representative images of Oil Red staining in TBT‐treated cells and control obtained with the Olympus CKX41 inverted phase contrast microscope under 200× magnification. Neutral lipids are stained red. Results are expressed as fold variation relative to the control group (dashed line). Error bars indicate mean ± S.E.M. (*n* = 6). *p < 0.05; **p < 0.01; ***p < 0.001.

In healthy adult human tissues, de novo fatty acid (FA) synthesis is usually suppressed, and the expression of lipogenic proteins is maintained at low levels [43]. Contrastingly, cancer cells display a metabolic rewiring, being able to synthesize large amounts of de novo FA and cholesterol [44], which is critical for maintaining cell membrane formation, rapid cancer cell growth and metastasis [44]. De novo FA synthesis mainly depends on two important enzymes, ACC and FASN (Figure 4A). ACC catalyzes the conversion of Ac‐CoA to malonyl‐CoA, the first limiting step in FA synthesis, whereas FASN synthesizes long‐chain saturated FA from malonyl‐CoA [44].

The effect of TBT (100 nM) on lipid metabolism of neoplastic prostate cells was investigated by analyzing several regulators of FA metabolism, namely, CD36, ACC, FASN, and CPT1A (Figure 4A).

The protein expression of the FA transporter CD36 significantly decreased in LNCaP cells upon TBT treatment (0.78‐fold variation to the control group, Figure 4B), with no significant changes found in PC3 cells (Figure 4B).

Considering ACC (Figure 4A), its expression augmented significantly in LNCaP cells after treatment with TBT (4.30 ± 0.64‐fold variation to the control group, Figure 4C); no significant changes were observed in TBT‐treated PC3 cells compared to the control (Figure 4C). TBT treatment significantly increased FASN (Figure 4A) expression levels in both tested cell lines, with 2.20 ± 0.14‐ and 1.70 ± 0.20‐fold variation to the control group, respectively, for LNCaP and PC3 cells (Figure 4D).

In the intracellular space, CPT1A is an enzymatic complex responsible for β‐oxidation, which allows the translocation of FA to the mitochondrial matrix. The products of lipid oxidation enter the Kreb's cycle to generate ATP (Figure 4A) [45]. In the neoplastic PC3 cells, exposure to TBT decreased CPT1A expression (0.72 ± 0.06‐fold variation to the control group, Figure 4E); no alterations were perceived in LNCaP cells (Figure 4E).

When intracellular FA content exceeds the cell's needs, it can be stored in reservoirs called lipid droplets (LDs) and recruited back by lipolysis in situations of scarcity [46]. Results of the Oil Red‐O assay showed that treatment with 100 nM TBT increased the relative number of LDs in LNCaP cells compared to the TBT‐untreated groups (1.21 ± 0.04‐fold variation, Figure 4F,G). However, in PC3 cells, the number of LDs decreased after TBT treatment (0.85 ± 0.02‐fold variation to the control group, Figure 4F,G).

### 3.5 TBT Stimulated Proliferation, Migration, and Invasion of Androgen‐Sensitive PCa Cells

Considering the deregulation observed in the metabolic pathways, androgen‐sensitive LNCaP cells were the cell line model selected to evaluate the effect of TBT on the proliferation, migration, and invasion capacity of PCa cells.

**FIGURE 5:**
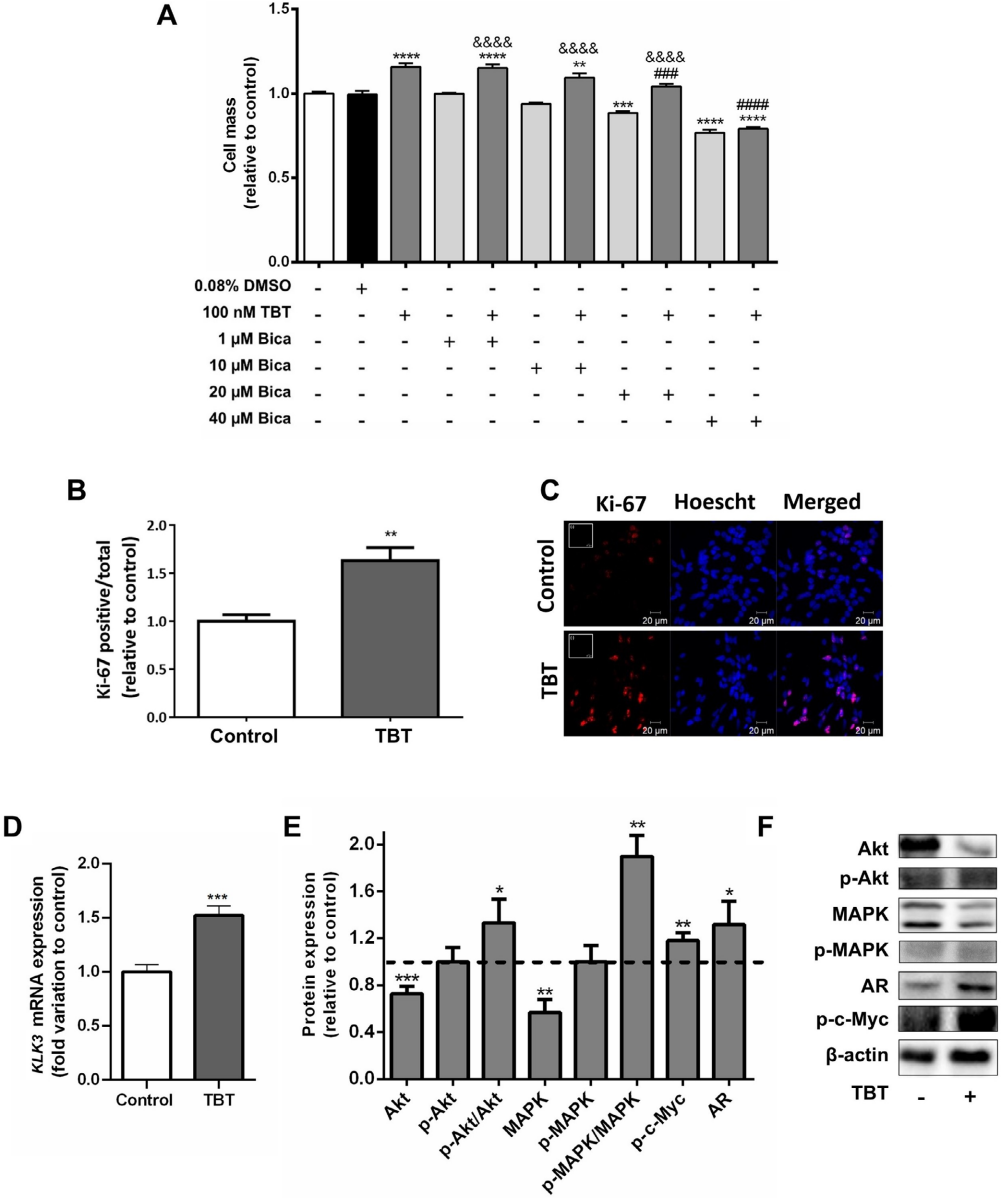
Effect of TBT on the proliferative activity of LNCaP cells. Cells were treated with 100 nM TBT for 48 h. (A) Percentage of cell mass in the presence or absence of Bicalutamide (Bica, 1–40 μM), estimated by the SRB assay. (B) Proliferation index determined by the immunofluorescence analysis of Ki‐67. Ten randomly selected fields per microscope cover glass were assessed. Data are expressed as the mean of Ki‐67‐positive cells relative to the total cell number. (C) Representative confocal microscopy images showing Ki‐67 labeling (red) and Hoechst 33342‐stained nuclei (blue) in TBT‐treated cells and control. Images were obtained with the Zeiss LSM 710 laser scanning confocal microscope under 630× magnification. Negative controls obtained by omission of the primary antibody are provided as insert panels (−). (D) mRNA expression of prostate‐specific antigen (PSA)‐encoding gene kallikrein‐related peptidase 3 (*KLK3*) determined by qPCR after normalization with β‐2‐microglobulin. (E) Protein expression of proliferation/survival regulators, Akt, p‐Akt, MAPK, p‐MAPK, p‐c‐Myc, and androgen receptor (AR) determined by WB analysis after normalization with β‐actin (hexaplicates). (F) Representative immunoblots. All results are expressed as fold variation relative to the control group (dashed line). Error bars indicate mean ± S.E.M. (*n* = 6). *p < 0.05, **p < 0.01, ***p < 0.001, ****p < 0.0001, when compared with the control group; ^###^ *p* < 0.001, ^####^ *p* < 0.0001, when compared with the TBT‐treated group; ^&&&&^ *p* < 0.0001, when compared with the respective bicalutamide group (same concentration).

The SRB assay and the immunofluorescent labeling of the nuclear proliferation marker Ki‐67 were used to assess LNCaP cell proliferation upon treatment with 100 nM TBT. It was observed that TBT increased cell proliferation as indicated by the SRB results (115.80 ± 12.12‐fold variation to the control group, Figure 5A) and the significantly augmented Ki‐67 labeling in TBT‐treated LNCaP cells compared to the control group (1.63 ± 0.14‐fold variation, Figure 5B,C).

TBT‐induced proliferative activity of LNCaP cells was attenuated in the presence of the AR inhibitor bicalutamide. As indicated by the SRB assay, the bicalutamide + TBT‐treated group displayed LNCaP cell proliferation reduced by ~12% or ~37% for 20 and 40 μM, respectively (Figure 5A), relative to TBT‐only. Bicalutamide alone (20 and 40 μM) also decreased LNCaP cell proliferation compared to the control group (88.42 ± 1.02‐ and 76.69 ± 1.78‐fold variation, respectively, Figure 5A).

TBT increased *KLK3* mRNA expression (1.52 ± 0.09‐fold variation to the control group, Figure 5D), with the analysis of protein expression of key molecular targets in cell proliferation/survival pathways showing that 100 nM TBT significantly increased AR and p‐c‐Myc levels in LNCaP‐treated cells when compared to the control group (1.65 ± 0.23‐ and 1.18 ± 0.04‐fold variation, respectively, Figure 5E). Inversely, Akt and MAPK expression was significantly lower in TBT‐treated LNCaP cells (0.54 ± 0.03‐ and 0.53 ± 0.08‐fold variation to the control group, Figure 5E), whereas the expression of their phosphorylated forms, respectively, p‐Akt and p‐MAPK, did not present significant differences. Nevertheless, the phosphorylated and total Akt and MAPK ratios were significantly increased upon TBT exposure (1.33 ± 0.25‐ and 1.90 ± 0.18‐fold variation to the control group, respectively, Figure 5E).

**FIGURE 6:**
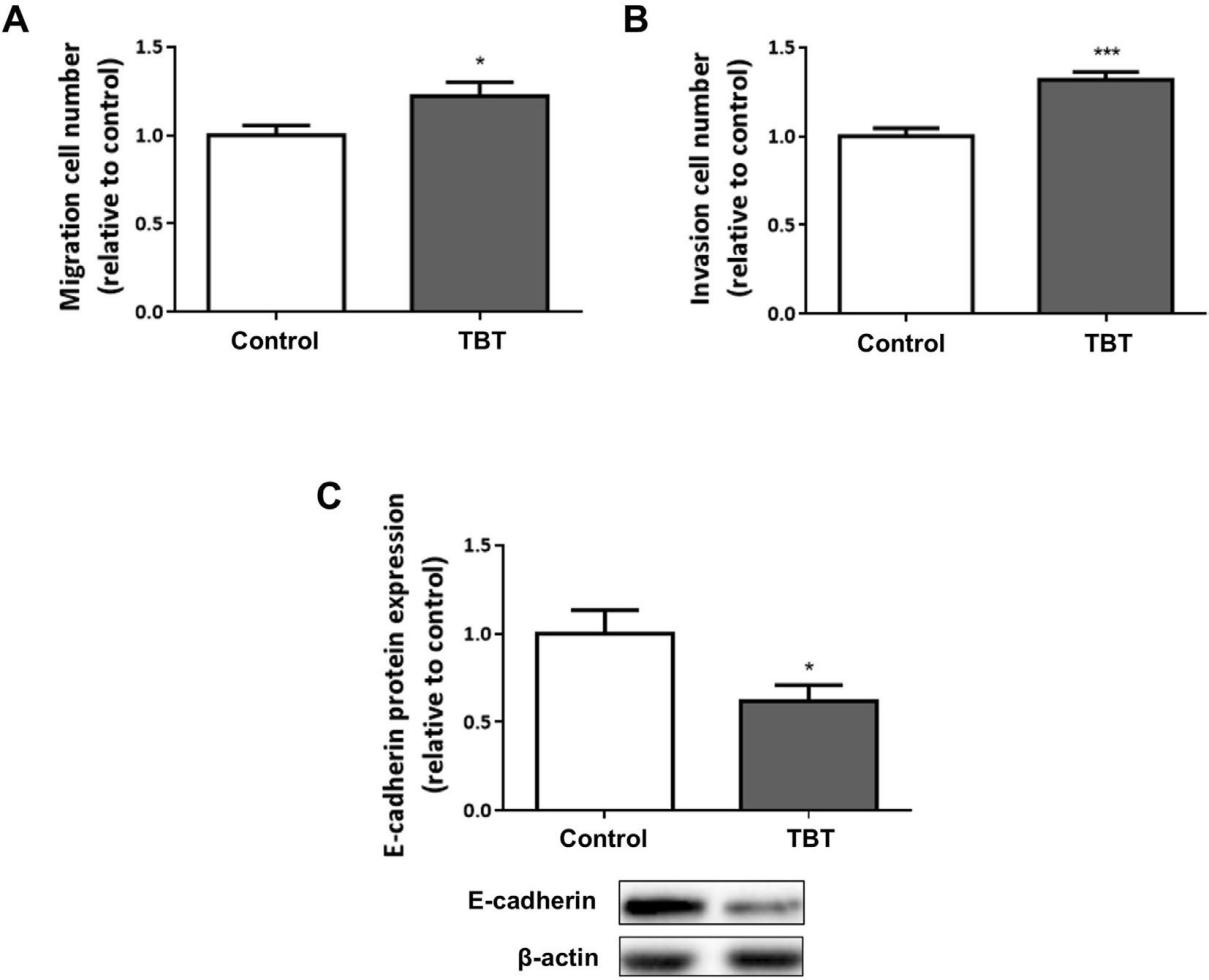
Effect of TBT on the migration and invasion activity of LNCaP cells. Cells were treated with 100 nM TBT for 24 (migration) or 48 (invasion) hours. (A) Migration and (B) invasion determined by transwell assays in uncoated or Matrigel‐coated chambers, respectively. Data are expressed as the mean number of migrating cells per 200× magnification field (10 fields were assessed for each experimental condition). (C) E‐cadherin expression determined by WB analysis after normalization with β‐actin (hexaplicates). Representative immunoblots are shown as bottom panels. All results are expressed as fold variation relative to the control group. Error bars indicate mean ± S.E.M. (*n* = 6). *p < 0.05; ***p < 0.001.

Tumor development, usually with the spreading of the disease to other tissues, relies on the activation of cancer cells' migratory and invasive capabilities, which depend on the epithelial–mesenchymal transition (EMT), a process characterized by the disruption of cell–cell adhesion, remodeling of the cytoskeleton, and changes in cell‐matrix adhesion [47, 48]. Transwell assays were used to assess migration and invasion of LNCaP cells in response to TBT. Treatment with 100 nM TBT significantly increased LNCaP cells' migration (1.22 ± 0.09‐fold variation to the control group, Figure 6A) and invasion (1.32 ± 0.04‐fold variation to the control group, Figure 6B).

E‐cadherin is a key molecule in cell‐to‐cell adhesion, and its suppressed levels are associated with PCa metastasis and chemoresistance [49, 50]. TBT‐treated LNCaP cells displayed a significantly decreased E‐cadherin expression compared to the control group (0.62 ± 0.09‐fold variation, Figure 6C).

### 3.6 Low TBT Concentration Maintains the Stimulatory Effects on LNCaP Cells' Viability, Proliferative Activity, and Migration/Invasion, Sustained by High LDL‐Cholesterol Availability and DHT

Substantial evidence shows that EDCs have damaging actions even at low concentrations [51, 52], which prompted us to investigate the effect of a 10× low TBT concentration (10 nM) on prostate cell fate. On the other hand, it is known that TBT is capable of interfering with lipid metabolism and androgenic signaling, and we previously showed that androgens (DHT, 10 nM) can enhance the effects of LDL (100 μg/mL) in increasing PCa cell viability, proliferation, and migration [16, 37, 51, 52]. These findings provided the rationale to evaluate the TBT effects plus 10 nM DHT or 100 μg/mL LDL.

**FIGURE 7:**
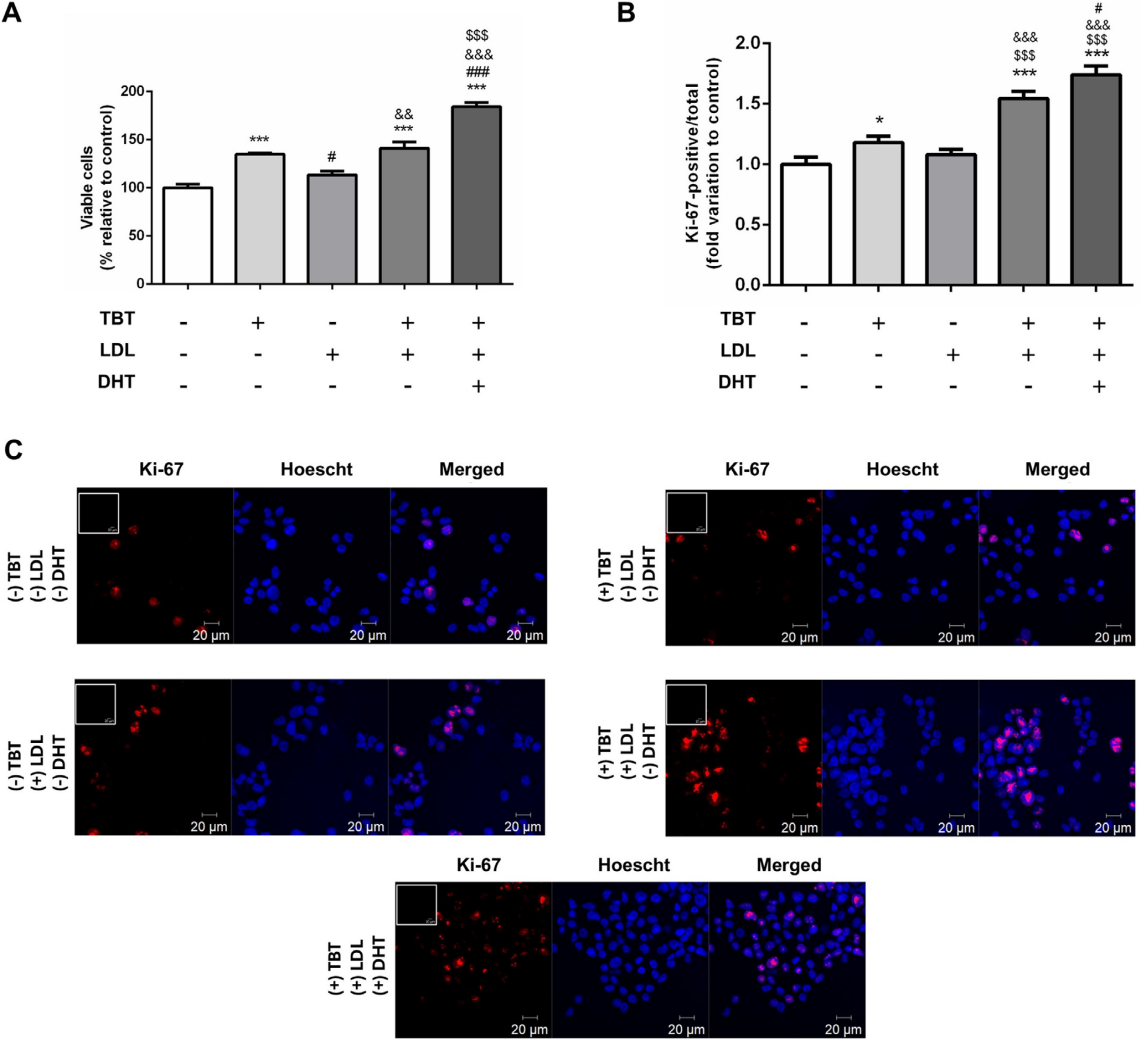
Effect of low TBT concentration on the viability and proliferative activity of LNCaP cells in the presence of LDL and DHT. Cells were treated with 10 nM TBT, 100 μg/mL LDL, and 10 nM DHT for 48 h. (A) Cell viability determined by the MTT assay. Results are expressed as % of the control group. (B) Proliferation index determined by the immunofluorescence analysis of Ki‐67. Ten randomly selected fields per microscope cover glass were assessed. Data are expressed as the mean of Ki67‐positive cells per total cell number relative to the control group. (C) Representative confocal microscopy images showing Ki‐67 labeling (red) and Hoechst 33342‐stained nuclei (blue) in the different experimental groups. Images were obtained with the Zeiss LSM 710 laser scanning confocal microscope under 630× magnification. Negative controls obtained by omission of the primary antibody are provided as insert panels (−). Error bars indicate mean ± S.E.M. (*n* = 6). *p < 0.05, **p < 0.01, ***p < 0.001, when compared with the control group; ^#^ *p* < 0.05 when compared with the TBT‐treated group; ^&^ *p* < 0.05, ^&&^ *p* < 0.01, ^&&&^ *p* < 0.001, when compared with the LDL‐treated group; ^$$$^ *p* < 0.001, when compared with the LDL + TBT‐treated group.

The results of the MTT assay showed that 10 nM TBT (lipid‐depleted FBS) increased LNCaP cells' viability by around 35% relative to the control group (Figure 7A). TBT's effect on cell viability was not affected by LDL supplementation, though it was intensified in the presence of DHT (1.35 ± 0.01‐ vs. 1.84 ± 0.04‐fold variation to control, Figure 7A).

LNCaP cell proliferation analyzed by Ki‐67 immunofluorescent labeling was significantly increased after 10 nM TBT treatment (1.18 ± 0.05‐fold variation to control, Figure 7B,C), which was enhanced by the presence of LDL or LDL + DHT (1.54 ± 0.06‐ and 1.74 ± 0.07‐fold variation to control, respectively, Figure 7B,C).

**FIGURE 8:**
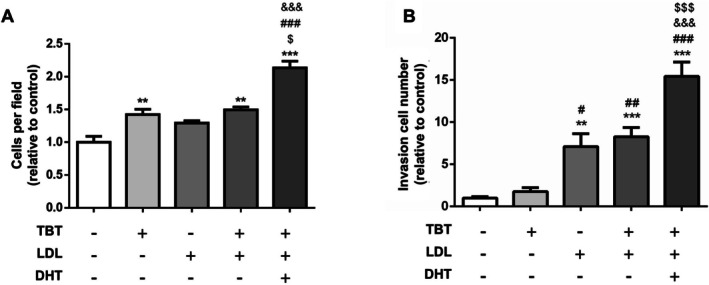
Effect of low TBT concentration on the migration and invasion activity of LNCaP cells in the presence of LDL and DHT. Cells were treated with 10 nM TBT, 100 μg/mL LDL, and 10 nM DHT for 48 h. (A) Migration and (B) invasion determined by transwell assays in uncoated or Matrigel‐coated chambers, respectively. Data are expressed as the mean number of migrating cells per 200× magnification field (five fields were assessed for each experimental condition). All results are expressed as fold variation relative to the control group. Error bars indicate mean ± S.E.M. (*n* = 6). **p < 0.01, ***p < 0.001, when compared with the control group; ^#^ *p* < 0.05, ^##^ *p* < 0.01, ^###^ *p* < 0.001, when compared with the TBT‐treated group; ^&&&^ *p* < 0.001, when compared with the LDL‐treated group; ^$^ *p* < 0.05, ^$$$^ *p* < 0.001, when compared with the LDL + TBT‐treated group.

Concerning migration, it was significantly increased in response to TBT treatment, regardless of the presence of LDL (1.42 ± 0.08‐ and 1.49 ± 0.05‐fold variation to control in TBT and TBT + LDL, respectively, Figure 8A). LNCaP cells treated simultaneously with TBT, LDL and DHT presented a marked increase in their migration capacity (2.14 ± 0.10‐fold variation to control, respectively, Figure 8A). No significant alterations were noticed in invasion upon TBT exposure (Figure 8B). Also, the significant increase observed in the invasion capacity of LDL‐treated cells was unchanged in the presence of TBT (7.11 ± 1.52‐ and 8.27 ± 1.11‐fold variation to control, respectively, Figure 8B). The addition of DHT exacerbated the LDL effect in promoting the invasion of LNCaP cells (15.43 ± 1.71‐fold variation to control, Figure 8B).

### 3.7 TBT Effects on LNCaP Cells' Lipid Metabolism Are Suppressed by LDL‐Cholesterol Availability

**FIGURE 9:**
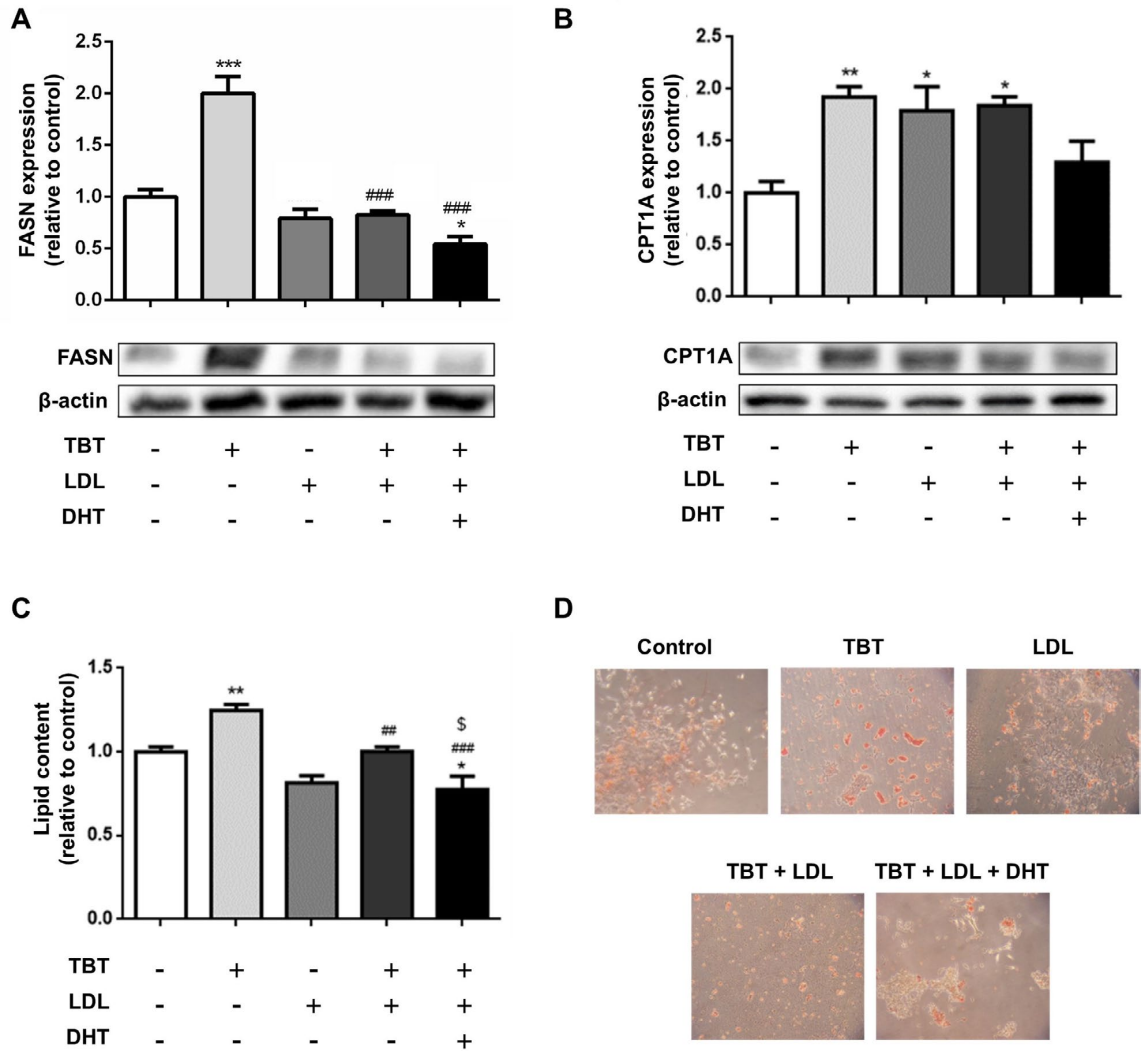
Effect of low TBT concentration in modulating lipid metabolism in LNCaP cells in the presence of LDL and DHT. Cells were treated with 10 nM TBT, 100 μg/mL LDL, and 10 nM DHT for 48 h. (A) FASN and (B) CPT1A expression determined by WB analysis after normalization with β‐actin (hexaplicates). Representative immunoblots are shown as bottom panels. (C) Lipid content determined by the Oil Red‐O assay. (D) Representative images of Oil Red‐O staining acquired with the Olympus CKX41 inverted phase contrast microscope at 200× magnification. All results are expressed as fold variation relative to the control group. Error bars indicate mean ± S.E.M. (*n* = 6). *p < 0.05, **p < 0.01, ***p < 0.001, when compared with the control group; ^##^ *p* < 0.01, ^###^ *p* < 0.001, when compared with the TBT‐treated group; ^$^ *p* < 0.05, when compared with the LDL + TBT‐treated group.

Exposure to TBT significantly increased FASN expression (2.00 ± 0.16‐fold variation to control, Figure 9A), which was suppressed by the presence of LDL, resuming the expression levels of the control (Figure 9A). The presence of DHT plus TBT + LDH further decreased FASN expression, being significant compared to the control group (0.54 ± 0.07‐fold variation, Figure 9A). Regarding CPT1A, there was a significant increase in its expression levels in TBT‐, LDH‐, and TBT + LDH‐treated cells (1.92 ± 0.10‐, 1.79 ± 0.23‐ and 1.84 ± 0.08‐fold variation to control, respectively, Figure 9B). The presence of DHT restored CPT1A expression levels in the TBT + LDH group to those of the control. It was also evaluated how TBT treatment and/or LDL/DHT availability modifies the capability of LNCaP cells for storing neutral lipids. Oil Red assay results showed that lipid content was significantly increased in TBT‐treated LNCaP cells (1.25 ± 0.04‐fold variation to control, Figure 9C,D). This effect was suppressed by LDL, which was further enhanced in the presence of DHT (1.00 ± 0.07‐fold variation to control in TBT + LDL vs. 0.77 ± 0.08‐fold variation to control in TBT + LDH + DHT, Figure 9C,D).

### 3.8 In Vivo TBT Treatment Modulated the Metabolic Status and Stimulated Cell Proliferation in Rat Prostate

**FIGURE 10:**
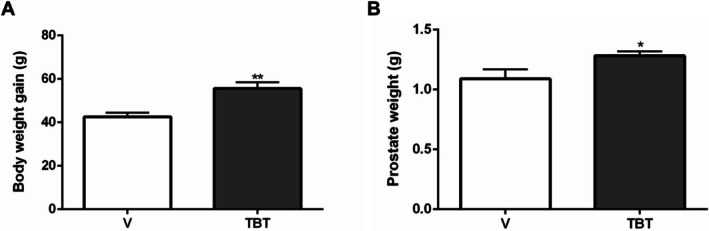
Effect of TBT on average (A) body weight gain and (B) prostate weight. TBT (50 μg/kg) was administered in vivo by oral gavage every 3 days for 45 days. Corn oil was used as vehicle (V). Error bars indicate mean ± S.E.M. (*n* = 7). *p < 0.05 and **p < 0.01.

TBT treatment (50 μg/kg) every 3 days for 45 days increased average rat body weight gain (55.57 ± 2.89 vs. 42.57 ± 1.94 g in the vehicle group, Figure 10A) and prostate weight (1.28 ± 0.04 vs. 1.09 ± 0.08 g in the vehicle group, Figure 10B).

**FIGURE 11:**
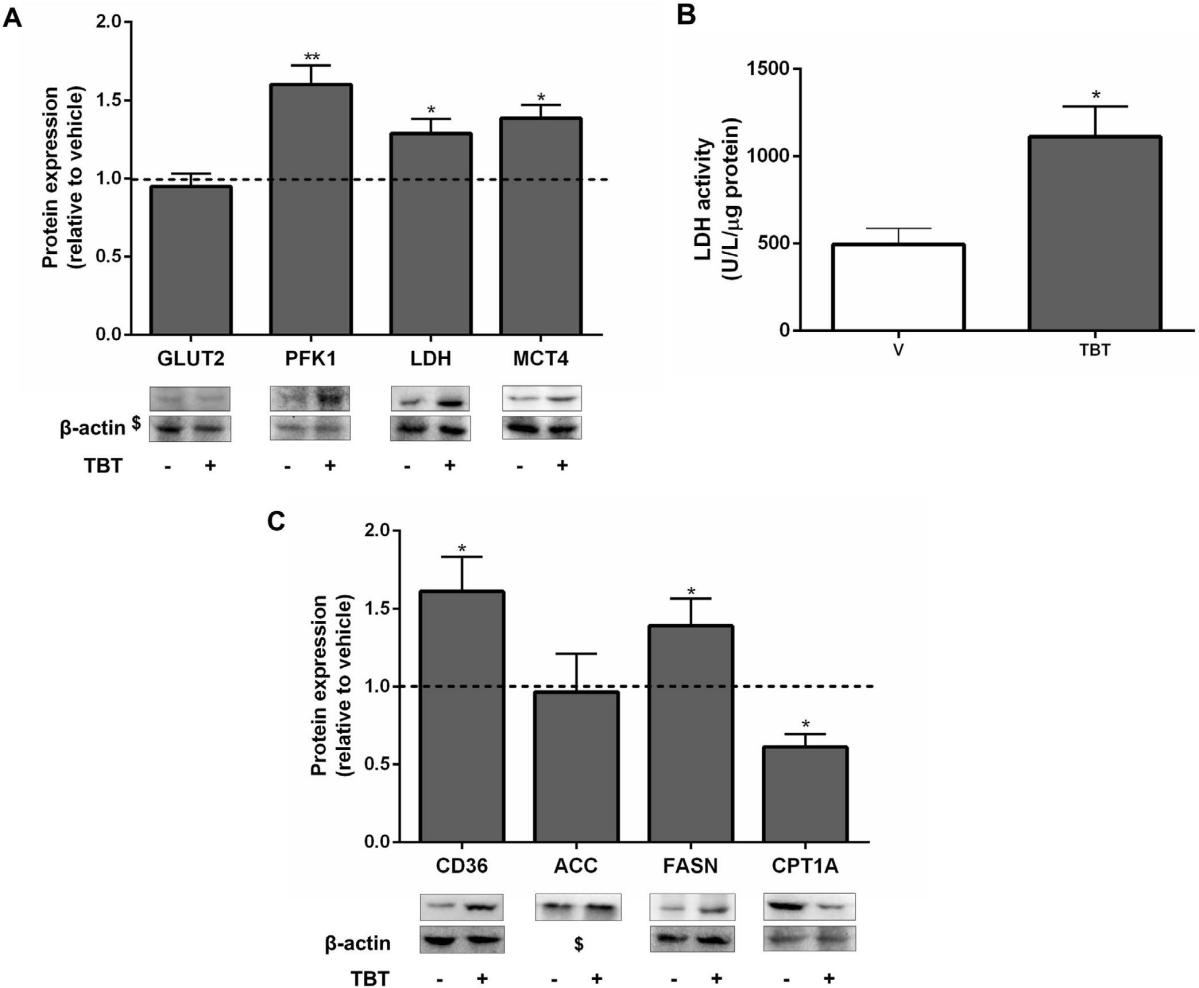
Impact of TBT on prostate glycolytic and lipid metabolism. TBT (50 μg/kg) was administered in vivo by oral gavage every 3 days for 45 days. Corn oil was used as vehicle (V). (A) Protein expression of GLUT2, PFK1, LDH, and MCT4 determined by WB analysis. (B) LDH enzymatic activity determined by spectrophotometric assay. (C) Protein expression of CD36, ACC, FASN, and CPT1A. WB results are expressed as fold variation relative to the V group (dashed line) after normalization with β‐actin (hexaplicates). $ GLUT2 and ACC were sequentially labeled on the same blot, with protein levels being quantified by normalization with the same β‐actin bands. Representative immunoblots are shown as bottom panels. Error bars indicate mean ± S.E.M. (*n* = 7). *p < 0.05 and **p < 0.01.

Changes in body and prostate weight due to TBT treatment were accompanied by altered expression of metabolic regulators in the rat prostate. Higher expression levels of PFK1, LDH, and MCT4 were observed in TBT‐treated animals compared to the vehicle group (1.60 ± 0.12‐, 1.29 ± 0.09‐, and 1.38 ± 0.09‐fold variation, respectively, Figure 11A), with no alterations perceived in GLUT2 expression. Concomitantly, exposure to TBT also increased LDH activity (1113.00 ± 173.80 U/L/μg protein vs. 496.20 ± 90.68 U/L/μg protein in the vehicle group, Figure 11B).

Regarding lipid metabolism, TBT increased the expression levels of the FA transporter CD36 (1.61 ± 0.22‐fold variation to the vehicle group, Figure 11C), while decreasing CPT1A expression (0.61 ± 0.08‐fold variation to the vehicle group, Figure 11C). TBT augmented the expression levels of FASN (1.39 ± 0.18‐fold variation to vehicle), with the ACC levels remaining unchanged (Figure 11C).

**FIGURE 12:**
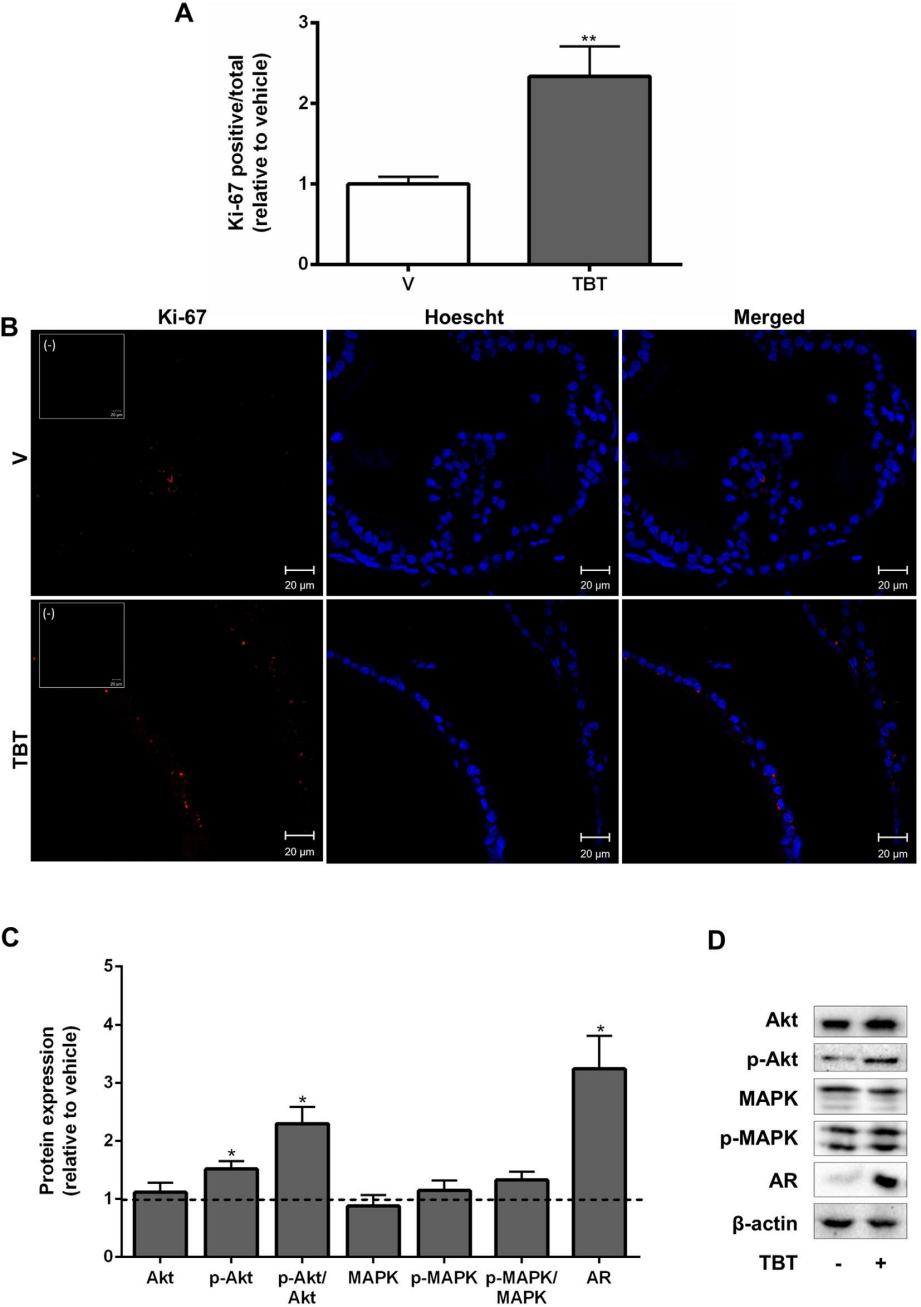
Effect of TBT in modulating the proliferative status of rat prostate. TBT (50 μg/kg) was administered in vivo by oral gavage every 3 days for 45 days. Corn oil was used as vehicle (V). (A) Proliferation index determined by the immunofluorescence analysis of Ki‐67. Data are expressed as the mean of Ki‐67‐positive cells relative to the total cell number as a percentage of the V. (B) Representative confocal microscopy images showing Ki‐67 labeling (red) and Hoechst 33342‐stained nuclei (blue) in TBT‐exposed and V prostates. Images were obtained with the Zeiss LSM 710 laser scanning confocal microscope under 630× magnification. Negative controls obtained by omission of the primary antibody are provided as insert panels (−). (E) Protein expression of proliferation/survival regulators Akt, p‐Akt, MAPK, p‐MAPK, and AR determined by WB analysis and normalized with β‐actin. (F) Representative immunoblots. Results are expressed as fold variation relative to the V group (dashed line). Error bars indicate mean ± S.E.M. (*n* = 7). *p < 0.05 and **p < 0.01.

Prostate proliferative status and the expression of proliferation/survival key regulators were also evaluated. TBT exposure increased the number of prostate Ki‐67‐positive cells by more than two‐fold relative to the vehicle group (Figure 12A,B). The enhanced labeling of Ki‐67 was underpinned by augmented expression levels of p‐Akt, as well as the p‐Akt/Akt ratio and AR (1.52 ± 0.14‐, 2.30 ± 0.30‐ and 3.24 ± 0.57‐fold variation to the vehicle group, respectively, Figure 12C). No differences were observed in the expression of Akt, MAPK, and p‐MAPK (Figure 12C).

## 4 Discussion

The present study investigated the effect of the EDC TBT on the viability, metabolic profile, proliferation, migration, and invasion of human prostate cells using in vitro and in vivo approaches.

The obtained results demonstrated that exposure to TBT increased the viability of androgen‐sensitive (LNCaP) and androgen‐insensitive (PC3) PCa cells (Figure 1). The TBT‐induced increase in PCa cell viability follows the observations in the literature, which have shown that EDCs with androgen‐like activity, as is the case of organotins, enhance the viability of PCa cells. For example, treatment with 100 nM TBT or 1 nM triphenyltin (TPT), another obesogenic triorganotin, increased LNCaP cell viability with the activation of AR‐mediated transcription [20].

Our next step was to explore the effect of TBT on the glycolytic metabolism of prostate cells. TBT treatment significantly increased glucose consumption and lactate production in the androgen‐sensitive LNCaP cells, which was underpinned by increased expression of PFK1 and MCT4 as well as increased LDH activity (Figure 2). Enhanced lactate production was also observed in TBT‐treated androgen‐insensitive PC3, likely due to the augmentation in LDH protein expression (Figure 2G) as MCT4 expression was decreased (Figure 2H). Nevertheless, MCT1 is another lactate transporter involved in lactate export, and both MCT1 and MCT4 were related to the progression of PCa and a poor prognosis of the disease [53, 54], which suggests MCT1 may be supporting TBT‐induced lactate export.

Despite the increase in glucose consumption (LNCaP) and lactate production (LNCaP and PC3 cells) with TBT treatment, GLUT2 expression was decreased in both PCa cell lines (Figure 2E). Augmented expression of GLUT2 has been described in cancer cells [55, 56]. However, in the case of PCa, other GLUTs have also been implicated in glucose handling [27, 57], which suggests that other GLUT isoforms can be responsible for the increased glucose entrance into LNCaP cells after TBT treatment. In the case of PC3 cells, as glucose consumption is not increased, we cannot exclude that the lactate being produced could have originated from glycogen or sources other than glucose. For example, from amino acids, through the conversion of alanine into glutamate and pyruvate by the activity of aminotransferase [58].

As far as we know, no other studies have investigated the TBT actions in modulating cancer cell metabolism. The present findings led us to believe that this EDC may change the plasticity of prostate cells toward a cancer progression phenotype. The results obtained in rat prostate in vivo followed those found in PCa cells, with exposure to TBT increasing the expression (PFK1, LDH, and MCT4) and activity (LDH) of glycolytic metabolism key players (Figure 11A,B), which indicates a disruption of the glycolytic flux toward a hyperglycolytic phenotype, similar to what occurs in cancerous cells.

The androgenic effect of TBT in regulating prostate cell glycolytic metabolism was investigated using the AR inhibitor bicalutamide. Indeed, bicalutamide attenuated the TBT‐induced enhancement of glucose consumption in the androgen‐sensitive LNCaP cells (Figure 3), which implicates the androgenic signaling in the TBT actions. This observation is reinforced by the fact that TBT did not significantly affect glucose consumption in the AR‐negative PC3 cells. Nevertheless, exposure to TBT increased the viability of PC3 cells and their lactate production, which supports the existence of AR‐independent mechanisms mediating TBT actions in this cell type.

Besides its androgenic properties, TBT is widely known as an obesogen, promoting the differentiation of adipocytes and increasing their size and number [17, 18]. Therefore, the influence of TBT in deregulating prostate cell lipid metabolism was investigated in vitro and in vivo. As expected, and confirming the effectiveness of gavage administration, TBT treatment increased rat body weight gain (Figure 10A). Alterations in lipid handling locally at the prostate level are also suggested by the altered expression of key transporters and enzymes. Our results demonstrated that TBT increased FASN expression in the rat prostate (Figure 11C) and enhanced ACC and FASN expression levels in LNCaP cells (Figure 4C,D). The upregulated expression and increased activity of both ACC and FASN are common features in many cancers, including PCa, and are strongly correlated with PCa progression [59, 60], which allows us to predict that TBT effects upregulating the expression of lipogenic enzymes and stimulating FA synthesis might potentially stimulate PCa development. CD36 is a major transporter responsible for FA uptake into cells, which is also overexpressed in PCa [61]. CD36 expression was upregulated in the prostate of TBT‐exposed animals (Figure 11C). However, a significant decrease in CD36 expression was observed in TBT‐treated LNCaP cells (Figure 4B). As previously mentioned, TBT has androgenic properties, and interestingly, our research group has shown reduced CD36 expression in LNCaP cells upon 48‐h androgenic stimulation [62]. The mechanisms responsible for the increased expression of CD36 in rat prostate in vivo are unknown. However, we may speculate that TBT increases CD36 expression indirectly by increasing FA availability in the tumor microenvironment, a well‐known feature of obesogenic dysregulation [10]. Unsaturated FA increased CD36 expression in human macrophages and rat fat and skeletal muscle tissue [63, 64]. However, in the case of the prostate, the relationship between FA availability and CD36 expression remains unknown. This is a relevant issue that must be exploited in studies dedicated to investigating the influence of TBT in other components of the tumor microenvironment and how it can impact prostate cells.

Concerning CPT1A, our results showed that TBT treatment decreased its protein expression in PC3 cells (Figure 4E) and rat prostates (Figure 11C), with no effect observed in LNCaP. These results are somewhat unexpected, as CPT1A is overexpressed in PCa compared to the benign tissues, specifically in high‐grade tumors [65], which supports the reported increased β‐oxidation in PCa cases [66]. Paradoxically, reduced CPT1A expression can also promote PCa progression through mechanisms related to metabolic reprogramming. It was reported that when the function of CPT1A is inhibited, the ability of cells to carry out β‐oxidation diminishes, which leads cancer cells to increase their reliance on glycolysis for energy production [67]. Moreover, reduced β‐oxidation enhances FA accumulation in the cell, potentially driving lipid biosynthesis pathways [68]. Our results, showing the upregulation of glycolytic flux in response to TBT and the enhanced expression of enzymes involved in de novo FA synthesis, are in line with both hypotheses.

In malignancy conditions, the excessive accumulation of esterified cholesterol in LDs was associated with the development of high‐grade prostate tumors and metastases [69]. Herein, we showed that exposure to TBT increased the lipid content (LDs) in LNCaP cells (Figure 4F). These results significantly point out TBT as a promoter of lipid storage and an inducer of lipid synthesis in neoplastic cell lines, in line with previous results of our research group and others concerning androgenic effects [20, 21, 37, 70]. Androgen stimulation of PCa cells was shown to increase the expression levels of FA synthesis enzymes, lipogenesis, and lipid content, mainly neutral lipids, phospholipids, and free cholesterol, which are stored in LDs and membranes, respectively [70]. Indeed, other authors have reported that TBT action results in the activation of AR‐mediated transcription in the LA16 clone established from LNCaP cells in a mechanism not dependent on the ligand binding [20]. Also, it was shown that this obesogen can increase AR expression, as reported in liver tissue and isolated hepatocytes [20, 21]. Accordingly, an increase in AR expression was found in LNCaP cells (Figure 5E) and rat prostate (Figure 12C) after TBT exposure. The fact that both AR and c‐Myc seem to regulate the expression of diverse metabolic proteins [24, 28], and c‐Myc enrichment is a signature of tumors expressing high levels of AR and vice versa [71], led us to investigate the protein levels of p‐c‐Myc. AR and p‐c‐Myc increased expression in response to TBT treatment (Figure 5E) supports that this could be a possible mechanism by which TBT influences metabolic enzymes expression and activity.

The ability of TBT in promoting PCa cell proliferation was previously described [20], but the underlying mechanisms are far from being completely understood. Our results demonstrated the growth‐promoting effects of TBT over prostate cells both in vitro and in vivo. TBT exposure enhanced the proliferative activity of LNCaP cells (Figure 5A–C), which supports its action as a tumor growth stimulator. Moreover, TBT treatment increased the proliferation index in rat prostate (Figure 12A,B), which was further confirmed by the observed augmentation of prostate weight (Figure 10B). These observations in the rat prostate upon in vivo treatment suggest that TBT may have a significant role in disturbing prostate tissue homeostasis and likely acts as a driving force in promoting the onset of PCa. Moreover, the bicalutamide suppression of the TBT‐induced LNCaP cell growth (Figure 5A), along with the higher *KLK3* mRNA expression in TBT‐treated LNCaP cells (Figure 5D), strongly suggests an overlap of the TBT action with AR‐dependent mechanisms. Nevertheless, the results obtained in the AR‐negative PC‐3 cells, with TBT increasing their viability and altering their metabolic profile, indicate that the actions of this EDC can also be mediated by the activation of androgen‐independent pathways.

Concerning the cell survival and proliferation pathways underlying the TBT‐induced proliferative status in prostate cells, the increased expression of AR (LNCaP cells and rat prostate) and p‐c‐Myc (LNCaP cells) is in line with the TBT proliferative effects. AR and c‐Myc are strongly associated with the regulation of cell cycle, survival and growth, having known roles in cancer progression and the establishment of the CRPC phenotype [71, 72, 73]. Moreover, the coordinated action of AR and c‐Myc in the development of PCa is also recognized [71].

TBT also targeted the phosphatidylinositol 3‐kinase (PI3K)/Akt and MAPK/extracellular signal‐regulated kinase (ERK) pathways in prostate cells. Although TBT did not affect the expression of p‐AKT and p‐MAPK in LNCaP cells (Figure 5E), the expression of these phosphorylated forms was enhanced relative to the total ones, as indicated by the increased p‐Akt/Akt and p‐MAPK/MAPK ratios (Figure 5E). Also, in the prostates of TBT‐treated animals, an augmented p‐Akt/Akt ratio was observed following increased p‐Akt expression levels (Figure 12C).

The stimulation of PI3K/Akt signaling is known to promote the survival of prostate tumor cells and prevent apoptosis [74], which supports the potential of TBT to drive tumor growth. To the best of our knowledge, this is the first study implicating PI3K/Akt and MAPK/ERK signaling in the TBT actions that enhance the proliferative status of prostate cells. Similar findings were found in breast cancer cells. TBT treatment increased p‐ERK levels with enhanced proliferative activity in MCF‐7 cells [75]. Even so, our results indicate that TBT might have oncogenic properties, stimulating the proliferation of PCa cells, which is probably also related to the activation of c‐Myc and AR‐mediated genomic mechanisms.

Besides promoting LNCaP cell viability and proliferation, exposure to TBT also increased migration and invasiveness (Figure 6A,B), suggesting that exposure to this EDC might favor metastasis development. The increased migration and invasiveness features of TBT‐treated LNCaP cells were supported by the diminished expression of the epithelial marker E‐cadherin (Figure 6C). Vimentin and N‐cadherin are mesenchymal cell markers usually displaying upregulated expression in the EMT process. However, their protein expression was undetectable (data not shown). Indeed, it has been described that LNCaP cells show low levels of vimentin [76, 77], being negative for N‐cadherin [78].

In line with the rationale that EDCs can induce damage even when exposure occurs at low concentrations, our results demonstrated that the effects of TBT in increasing LNCaP cell viability, proliferation and migration were maintained even at a 10× lower concentration and lipid‐depleted conditions. These findings confirm the need for awareness of TBT exposure and its relationship with PCa, as well as for further investigation of the mechanisms of action and biological processes affected by this compound. Noteworthy, the dual presence of LDL and DHT enhanced the pro‐survival and aggressiveness features of TBT‐treated PCa cells (Figure 7A,B). These results are in line with our and others' previous studies demonstrating that the presence of androgens potentiates the effects of LDL on prostate cells, which was supported by the evidence that the androgens increased LDL uptake [37, 70, 79, 80, 81]. On the other hand, cholesterol, as the precursor for steroidogenesis, has been pointed to fuel intratumoral androgen synthesis, accelerating the growth of prostate tumors, with the inhibition of LDL receptor being able to sensitize PCa cells to chemotherapeutic drugs [79, 82, 83]. Androgens have been shown to stimulate lipid uptake, synthesis, and storage, as well as lipolysis from LDs, by interacting with the transcriptional machinery and thus regulating the gene expression network [84, 85, 86]. Besides gaining awareness of the TBT actions favoring PCa development, our experimental outcomes shed light on the fact that lipid availability and an androgenic hormonal milieu may exacerbate the TBT effects.

Concerning the effects of TBT on lipid handling, the 10 nM concentration also retained the ability to increase FASN expression and lipid content (Figure 9). Though not affecting the effect of TBT on CPT1A, the presence of LDL restored FASN expression levels to those of the control with an accompanying decrease in lipid content. Therefore, it is liable to assume that in lipid‐depleted conditions, TBT sustains de novo lipid synthesis to fulfill cell energy requirements. Contrarily, LNCaP cells did not prioritize lipogenesis under high LDL availability, apparently maintaining similar levels of β‐oxidation (unaltered CPT1A expression relative to lipid‐depleted conditions) sustained by exogenous lipids (LDL). In this case, the presence of DHT did not change LDL actions over lipid handling in TBT‐treated LNCaP cells.

Finally, our results support the potential pro‐cancer effects of TBT, which, due to its obesogenic and androgenic activity, may disrupt prostate cell metabolism and promote PCa cell viability, proliferation, and migration/invasion. Further studies using established carcinogenesis models (e.g., xenografts, chemically induced carcinogenesis) would confirm whether TBT can accelerate cancer progression.

Besides highlighting the relevance of EDCs contributing to PCa development, the present findings are particularly worrying, considering that the TBT's effects are maintained even at low concentrations and can be exacerbated by lipid availability and the androgenic hormonal milieu. Moreover, despite TBT use being banned in 2008, an extensive list of TBT‐based antifouling products is still available in the market of several countries [87], and TBT is characterized by its environmental persistence [88, 89]. Therefore, TBT remains a current environmental hazard, which, in light of these results, would signify a relevant public health problem in several coastal areas worldwide where TBT's hot‐spot pollution sites have been identified [24].

## 5 Conclusions

The present study shows TBT as a potential inducer of PCa progression and aggressiveness and contributes to increasing awareness about the roles of EDCs in the prostate carcinogenic process. Moreover, our findings may suggest that exposure to EDCs contributes to the complex intricacy of managing and treating this human neoplasia. Also, they provide the basis for investigating the TBT actions over other human cancers, namely, those dependent on hormonal stimulation and for which obesity is a major risk factor.

## Author Contributions

**Mariana Feijó, Luís P. Brás,** and **Catarina M. D. Serra:** writing – original draft, methodology, investigation, and writing – review and editing. **Lara R. S. Fonseca:** writing – review and editing, methodology, and investigation. **Sara Correia:** writing – review and editing, methodology, and supervision. **Bruno J. Pereira:** writing – review and editing and formal analysis. **Ana P. Duarte:** writing – review and editing, project administration, and funding acquisition. **Henrique J. Cardososss** and **Cátia V. Vaz:** writing – review and editing, methodology, supervision, and conceptualization. **Sílvia Socorro:** writing – review and editing, validation, methodology, supervision, project administration, funding acquisition, formal analysis, and conceptualization.

## Funding

This work was supported by the Fundação para a Ciência e a Tecnologia (M.F. PhD fellowship 2021.07367.BD, L.R.S.F. PhD fellowship 2021.07634.BD, and UIDB/00709/2020), and the European Regional Development Fund (POCI‐01‐0145‐FEDER‐029114).

## Conflicts of Interest

The authors declare no conflicts of interest.

## Data Availability

The data that support the findings of this study are available from the corresponding author upon reasonable request.

## References

[1] E. D. Crawford , “Epidemiology of Prostate Cancer,” Urology 62, no. 6 (2003): 3–12. 10.1016/j.urology.2003.10.013 14706503

[2] S. R. Denmeade , X. S. Lin , and J. T. Isaacs , “Role of Programmed (Apoptotic) Cell Death During the Progression and Therapy for Prostate Cancer,” Prostate 28, no. 4 (1996): 251–265. 8602401 10.1002/(SICI)1097-0045(199604)28:4<251::AID-PROS6>3.0.CO;2-G

[3] J. D. Debes and D. J. Tindall , “The Role of Androgens and the Androgen Receptor in Prostate Cancer,” Cancer Letters 187, no. 1–2 (2002): 1–7. 12359344 10.1016/s0304-3835(02)00413-5

[4] S. Wu , S. Powers , W. Zhu , and Y. A. Hannun , “Substantial Contribution of Extrinsic Risk Factors to Cancer Development,” Nature 529, no. 7584 (2016): 43–47. 26675728 10.1038/nature16166 PMC4836858

[5] E. R. Kabir , M. S. Rahman , and I. Rahman , “A Review on Endocrine Disruptors and Their Possible Impacts on Human Health,” Environmental Toxicology and Pharmacology 40, no. 1 (2015): 241–258. 26164742 10.1016/j.etap.2015.06.009

[6] A. M. Soto and C. Sonnenschein , “Environmental Causes of Cancer: Endocrine Disruptors as Carcinogens,” Nature Reviews. Endocrinology 6, no. 7 (2010): 363–370. 10.1038/nrendo.2010.87 PMC3933258 20498677

[7] E. Haverinen , M. F. Fernandez , V. Mustieles , and H. Tolonen , “Metabolic Syndrome and Endocrine Disrupting Chemicals: An Overview of Exposure and Health Effects,” International Journal of Environmental Research and Public Health 18, no. 24 (2021): 13047. 34948652 10.3390/ijerph182413047 PMC8701112

[8] D. Hanahan , “Hallmarks of Cancer: New Dimensions,” Cancer Discovery 12, no. 1 (2022): 31–46. 35022204 10.1158/2159-8290.CD-21-1059

[9] F. Grün and B. Blumberg , “Environmental Obesogens: Organotins and Endocrine Disruption via Nuclear Receptor Signaling,” Endocrinology 147, no. 6 (2006): S50–S55. 16690801 10.1210/en.2005-1129

[10] R. A. Taylor , J. Lo , N. Ascui , and M. J. Watt , “Linking Obesogenic Dysregulation to Prostate Cancer Progression,” Endocrine Connections 4, no. 4 (2015): R68–R80. 26581226 10.1530/EC-15-0080 PMC4653354

[11] P. D. Darbre , “How Could Endocrine Disrupters Affect Human Health?,” in Endocrine Disruption and Human Health, ed. P. D. Darbre (Elsevier, 2015), 27–45.

[12] R. Lauretta , A. Sansone , M. Sansone , F. Romanelli , and M. Appetecchia , “Endocrine Disrupting Chemicals: Effects on Endocrine Glands,” Frontiers in Endocrinology 10 (2019): 178. 30984107 10.3389/fendo.2019.00178 PMC6448049

[13] Endocrine Society , “Where We Stand on EDCs,” accessed September 16, 2024, https://www.endocrine.org/topics/edc/where‐we‐stand.

[14] M. Champ , “New IMO Convention to Control Anti‐Fouling Systems on Ships,” Sea Technology 42 (2001): 48–50.

[15] J. Beyer , Y. Song , K. E. Tollefsen , et al., “The Ecotoxicology of Marine Tributyltin (TBT) Hotspots: A Review,” Marine Environmental Research 179 (2022): 105689. 35777303 10.1016/j.marenvres.2022.105689

[16] A. Pereira‐Fernandes , C. Vanparys , T. L. M. Hectors , et al., “Unraveling the Mode of Action of an Obesogen: Mechanistic Analysis of the Model Obesogen Tributyltin in the 3T3‐L1 Cell Line,” Molecular and Cellular Endocrinology 370, no. 1–2 (2013): 52–64. 23428407 10.1016/j.mce.2013.02.011

[17] R. Chamorro‐García , M. Sahu , R. J. Abbey , J. Laude , N. Pham , and B. Blumberg , “Transgenerational Inheritance of Increased Fat Depot Size, Stem Cell Reprogramming, and Hepatic Steatosis Elicited by Prenatal Exposure to the Obesogen Tributyltin in Mice,” Environmental Health Perspectives 121, no. 3 (2013): 359–366. 23322813 10.1289/ehp.1205701 PMC3621201

[18] E. Lutfi , N. Riera‐Heredia , M. Córdoba , et al., “Tributyltin and Triphenyltin Exposure Promotes In Vitro Adipogenic Differentiation but Alters the Adipocyte Phenotype in Rainbow Trout,” Aquatic Toxicology 188 (2017): 148–158. 28527383 10.1016/j.aquatox.2017.05.001

[19] H. Guo , H. Yan , D. Cheng , X. Wei , R. Kou , and J. Si , “Tributyltin Exposure Induces Gut Microbiome Dysbiosis With Increased Body Weight Gain and Dyslipidemia in Mice,” Environmental Toxicology and Pharmacology 60 (2018): 202–208. 29738946 10.1016/j.etap.2018.04.020

[20] Y. Yamabe , A. Hoshino , N. Imura , T. Suzuki , and S. Himeno , “Enhancement of Androgen‐Dependent Transcription and Cell Proliferation by Tributyltin and Triphenyltin in Human Prostate Cancer Cells,” Toxicology and Applied Pharmacology 169, no. 2 (2000): 177–184. 11097870 10.1006/taap.2000.9067

[21] A. S. Mortensen and A. Arukwe , “Modulation of Xenobiotic Biotransformation System and Hormonal Responses in Atlantic Salmon (*Salmo salar*) After Exposure to Tributyltin (TBT),” Comparative Biochemistry and Physiology Part C, Toxicology & Pharmacology 145, no. 3 (2007): 431–441. 17344101 10.1016/j.cbpc.2007.01.013

[22] A. S. Mortensen and A. Arukwe , “Effects of Tributyltin (TBT) on In Vitro Hormonal and Biotransformation Responses in Atlantic Salmon ( *Salmo salar* ),” Journal of Toxicology and Environmental Health. Part A 72, no. 3–4 (2009): 209–218. 19184735 10.1080/15287390802539061

[23] P. D. Darbre , “Endocrine Disruptors and Obesity,” Current Obesity Reports 6, no. 1 (2017): 18–27. 28205155 10.1007/s13679-017-0240-4 PMC5359373

[24] H. J. Cardoso , T. M. A. Carvalho , L. R. S. Fonseca , M. I. Figueira , C. V. Vaz , and S. Socorro , “Revisiting Prostate Cancer Metabolism: From Metabolites to Disease and Therapy,” Medicinal Research Reviews 41, no. 3 (2021): 1499–1538. 33274768 10.1002/med.21766

[25] J. S. Horoszewicz , S. S. Leong , E. Kawinski , et al., “LNCaP Model of Human Prostatic Carcinoma,” Cancer Research 43, no. 4 (1983): 1809–1818. 6831420

[26] M. Kaighn , K. S. Narayan , Y. Ohnuki , J. F. Lechner , and L. W. Jones , “Establishment and Characterization of a Human Prostatic Carcinoma Cell Line (PC‐3),” Investigative Urology 17, no. 1 (1979): 16–23. 447482

[27] C. V. Vaz , M. G. Alves , R. Marques , et al., “Androgen‐Responsive and Nonresponsive Prostate Cancer Cells Present a Distinct Glycolytic Metabolism Profile,” International Journal of Biochemistry & Cell Biology 44, no. 11 (2012): 2077–2084. 22964025 10.1016/j.biocel.2012.08.013

[28] C. V. Vaz , R. Marques , M. G. Alves , et al., “Androgens Enhance the Glycolytic Metabolism and Lactate Export in Prostate Cancer Cells by Modulating the Expression of GLUT1, GLUT3, PFK, LDH and MCT4 Genes,” Journal of Cancer Research and Clinical Oncology 142, no. 1 (2016): 5–16. 26048031 10.1007/s00432-015-1992-4 PMC11819079

[29] K. Kannan , K. Senthilkumar , and J. P. Giesy , “Occurrence of Butyltin Compounds in Human Blood,” Environmental Science & Technology 33, no. 10 (1999): 1776–1779.

[30] K. Ishida , K. Aoki , T. Takishita , et al., “Low‐Concentration Tributyltin Decreases GluR2 Expression via Nuclear Respiratory Factor‐1 Inhibition,” International Journal of Molecular Sciences 18, no. 8 (2017): 1754. 28800112 10.3390/ijms18081754 PMC5578144

[31] M. Penza , M. Jeremic , E. Marrazzo , et al., “The Environmental Chemical Tributyltin Chloride (TBT) Shows Both Estrogenic and Adipogenic Activities in Mice Which Might Depend on the Exposure Dose,” Toxicology and Applied Pharmacology 255, no. 1 (2011): 65–75. 21683088 10.1016/j.taap.2011.05.017

[32] J. Xu , K. Ou , C. Chen , et al., “Tributyltin Exposure Disturbs Hepatic Glucose Metabolism in Male Mice,” Toxicology 425 (2019): 152242. 31306684 10.1016/j.tox.2019.152242

[33] A. Mehlem , C. E. Hagberg , L. Muhl , U. Eriksson , and A. Falkevall , “Imaging of Neutral Lipids by Oil Red O for Analyzing the Metabolic Status in Health and Disease,” Nature Protocols 8, no. 6 (2013): 1149–1154. 23702831 10.1038/nprot.2013.055

[34] N. N. Pavlova and C. B. Thompson , “The Emerging Hallmarks of Cancer Metabolism,” Cell Metabolism 23, no. 1 (2016): 27–47. 26771115 10.1016/j.cmet.2015.12.006 PMC4715268

[35] S. M. Green , E. A. Mostaghel , and P. S. Nelson , “Androgen Action and Metabolism in Prostate Cancer,” Molecular and Cellular Endocrinology 360, no. 1–2 (2012): 3–13. 22453214 10.1016/j.mce.2011.09.046 PMC4124858

[36] H. J. Cardoso , M. I. Figueira , C. V. Vaz , et al., “Glutaminolysis Is a Metabolic Route Essential for Survival and Growth of Prostate Cancer Cells and a Target of 5α‐Dihydrotestosterone Regulation,” Cellular Oncology 44, no. 2 (2021): 385–403. 33464483 10.1007/s13402-020-00575-9 PMC12980676

[37] H. J. Cardoso , M. I. Figueira , T. M. Carvalho , et al., “Androgens and Low Density Lipoprotein‐Cholesterol Interplay in Modulating Prostate Cancer Cell Fate and Metabolism,” Pathology, Research and Practice 240 (2022): 154181. 36327818 10.1016/j.prp.2022.154181

[38] C. Y. Mah , Z. D. Nassar , J. V. Swinnen , and L. M. Butler , “Lipogenic Effects of Androgen Signaling in Normal and Malignant Prostate,” Asian Journal of Urology 7, no. 3 (2020): 258–270. 32742926 10.1016/j.ajur.2019.12.003 PMC7385522

[39] F. Nualart , M. Garcia , R. Medina , and G. Owen , “Glucose Transporters in Sex Steroid Hormone Related Cancer,” Current Vascular Pharmacology 7, no. 4 (2009): 534–548. 19485886 10.2174/157016109789043928

[40] A. P. Halestrap and M. C. Wilson , “The Monocarboxylate Transporter Family—Role and Regulation,” IUBMB Life 64, no. 2 (2012): 109–119. 22162139 10.1002/iub.572

[41] D. Mishra and D. Banerjee , “Lactate Dehydrogenases as Metabolic Links Between Tumor and Stroma in the Tumor Microenvironment,” Cancers 11, no. 6 (2019): 750. 31146503 10.3390/cancers11060750 PMC6627402

[42] M. V. Liberti and J. W. Locasale , “The Warburg Effect: How Does It Benefit Cancer Cells?,” Trends in Biochemical Sciences 41, no. 3 (2016): 211–218. 29482833 10.1016/j.tibs.2016.01.004

[43] F. Ameer , L. Scandiuzzi , S. Hasnain , H. Kalbacher , and N. Zaidi , “De Novo Lipogenesis in Health and Disease,” Metabolism 63, no. 7 (2014): 895–902. 24814684 10.1016/j.metabol.2014.04.003

[44] Q. Liu , Q. Luo , A. Halim , and G. Song , “Targeting Lipid Metabolism of Cancer Cells: A Promising Therapeutic Strategy for Cancer,” Cancer Letters 401 (2017): 39–45. 28527945 10.1016/j.canlet.2017.05.002

[45] T. M. Carvalho , H. J. Cardoso , M. I. Figueira , C. V. Vaz , and S. Socorro , “The Peculiarities of Cancer Cell Metabolism: A Route to Metastasization and a Target for Therapy,” European Journal of Medicinal Chemistry 171 (2019): 343–363. 30928707 10.1016/j.ejmech.2019.03.053

[46] T. Petan , E. Jarc , and M. Jusović , “Lipid Droplets in Cancer: Guardians of Fat in a Stressful World,” Molecules 23, no. 8 (2018): 1941. 30081476 10.3390/molecules23081941 PMC6222695

[47] N. A. Bhowmick and H. L. Moses , “Tumor–Stroma Interactions,” Current Opinion in Genetics & Development 15, no. 1 (2005): 97–101. 15661539 10.1016/j.gde.2004.12.003 PMC2819733

[48] V. Gkretsi and T. Stylianopoulos , “Cell Adhesion and Matrix Stiffness: Coordinating Cancer Cell Invasion and Metastasis,” Frontiers in Oncology 8 (2018): 145. 29780748 10.3389/fonc.2018.00145 PMC5945811

[49] A. P. Putzke , A. P. Ventura , A. M. Bailey , et al., “Metastatic Progression of Prostate Cancer and e‐Cadherin: Regulation by Zeb1 and Src Family Kinases,” American Journal of Pathology 179, no. 1 (2011): 400–410. 21703419 10.1016/j.ajpath.2011.03.028 PMC3123858

[50] W. Wang , L. Wang , A. Mizokami , et al., “Down‐Regulation of E‐Cadherin Enhances Prostate Cancer Chemoresistance via Notch Signaling,” Chinese Journal of Cancer 36, no. 1 (2017): 1–13. 28356132 10.1186/s40880-017-0203-x PMC5372329

[51] L. N. Vandenberg , T. Colborn , T. B. Hayes , et al., “Hormones and Endocrine‐Disrupting Chemicals: Low‐Dose Effects and Nonmonotonic Dose Responses,” Endocrine Reviews 33, no. 3 (2012): 378–455. 22419778 10.1210/er.2011-1050 PMC3365860

[52] L. N. Vandenberg , “Low‐Dose Effects of Hormones and Endocrine Disruptors,” Vitamins and Hormones 94 (2014): 129–165. 24388189 10.1016/B978-0-12-800095-3.00005-5

[53] S. Andersen , Ø. Solstad , L. Moi , et al., “Organized Metabolic Crime in Prostate Cancer: The Coexpression of MCT1 in Tumor and MCT4 in Stroma Is an Independent Prognosticator for Biochemical Failure,” in Urologic Oncology: Seminars and Original Investigations, vol. 33 (Elsevier, 2015), 338.e9–338.e17. 10.1016/j.urolonc.2015.05.013 26066969

[54] N. Pértega‐Gomes , J. R. Vizcaíno , V. Miranda‐Gonçalves , et al., “Monocarboxylate Transporter 4 (MCT4) and CD147 Overexpression Is Associated With Poor Prognosis in Prostate Cancer,” BMC Cancer 11, no. 1 (2011): 1–9. 10.1186/1471-2407-11-312 PMC3157459 21787388

[55] A. Giatromanolaki , E. Sivridis , S. Arelaki , and M. I. Koukourakis , “Expression of Enzymes Related to Glucose Metabolism in Non‐Small Cell Lung Cancer and Prognosis,” Experimental Lung Research 43, no. 4–5 (2017): 167–174. 28644754 10.1080/01902148.2017.1328714

[56] I. Hamann , D. Krys , D. Glubrecht , et al., “Expression and Function of Hexose Transporters GLUT1, GLUT2, and GLUT5 in Breast Cancer—Effects of Hypoxia,” FASEB Journal 32, no. 9 (2018): 5104–5118. 29913554 10.1096/fj.201800360R

[57] J. D. Chandler , E. D. Williams , J. L. Slavin , J. D. Best , and S. Rogers , “Expression and Localization of GLUT1 and GLUT12 in Prostate Carcinoma,” CA: A Cancer Journal for Clinicians 97, no. 8 (2003): 2035–2042. 10.1002/cncr.11293 12673735

[58] M. Kim , J. Gwak , S. Hwang , S. Yang , and S. M. Jeong , “Mitochondrial GPT2 Plays a Pivotal Role in Metabolic Adaptation to the Perturbation of Mitochondrial Glutamine Metabolism,” Oncogene 38, no. 24 (2019): 4729–4738. 30765862 10.1038/s41388-019-0751-4

[59] M. Huang , A. Koizumi , S. Narita , et al., “Diet‐Induced Alteration of Fatty Acid Synthase in Prostate Cancer Progression,” Oncogene 5, no. 2 (2016): e195. 10.1038/oncsis.2015.42 PMC5154344 26878389

[60] Z. Cao , Y. Xu , F. Guo , et al., “FASN Protein Overexpression Indicates Poor Biochemical Recurrence‐Free Survival in Prostate Cancer,” Disease Markers 2020 (2020): 1–9. 10.1155/2020/3904947 PMC7321525 32655718

[61] M. J. Watt , A. K. Clark , L. A. Selth , et al., “Suppressing Fatty Acid Uptake Has Therapeutic Effects in Preclinical Models of Prostate Cancer,” Science Translational Medicine 11, no. 478 (2019): eaau5758. 30728288 10.1126/scitranslmed.aau5758

[62] H. J. M. M. M. Cardoso , Prostate Cancer Cells Metabolism on the Interplay of Androgenic Regulation and Metabolic Environment (Universidade da Beira Interior, 2020).

[63] R. Fabris , E. Nisoli , A. M. Lombardi , et al., “Preferential Channeling of Energy Fuels Toward Fat Rather Than Muscle During High Free Fatty Acid Availability in Rats,” Diabetes 50, no. 3 (2001): 601–608. 11246880 10.2337/diabetes.50.3.601

[64] J.‐C. Vallvé , K. Uliaque , J. Girona , et al., “Unsaturated Fatty Acids and Their Oxidation Products Stimulate CD36 Gene Expression in Human Macrophages,” Atherosclerosis 164, no. 1 (2002): 45–56. 12119192 10.1016/s0021-9150(02)00046-1

[65] T. W. Flaig , M. Salzmann‐Sullivan , L. J. Su , et al., “Lipid Catabolism Inhibition Sensitizes Prostate Cancer Cells to Antiandrogen Blockade,” Oncotarget 8, no. 34 (2017): 56051–56065. 28915573 10.18632/oncotarget.17359 PMC5593544

[66] Y. Liu , “Fatty Acid Oxidation Is a Dominant Bioenergetic Pathway in Prostate Cancer,” Prostate Cancer and Prostatic Diseases 9, no. 3 (2006): 230–234. 16683009 10.1038/sj.pcan.4500879

[67] I. R. Schlaepfer , L. M. Glodé , C. A. Hitz , et al., “Inhibition of Lipid Oxidation Increases Glucose Metabolism and Enhances 2‐Deoxy‐2‐[18 F]Fluoro‐d‐Glucose Uptake in Prostate Cancer Mouse Xenografts,” Molecular Imaging and Biology 17 (2015): 529–538. 25561013 10.1007/s11307-014-0814-4 PMC4493937

[68] K. N. Frayn , Metabolic Regulation: A Human Perspective (John Wiley & Sons, 2013).

[69] S. Yue , J. Li , S. Y. Lee , et al., “Cholesteryl Ester Accumulation Induced by PTEN Loss and PI3K/AKT Activation Underlies Human Prostate Cancer Aggressiveness,” Cell Metabolism 19, no. 3 (2014): 393–406. 24606897 10.1016/j.cmet.2014.01.019 PMC3969850

[70] K. D. Tousignant , A. Rockstroh , A. Taherian Fard , et al., “Lipid Uptake Is an Androgen‐Enhanced Lipid Supply Pathway Associated With Prostate Cancer Disease Progression and Bone Metastasis,” Molecular Cancer Research 17, no. 5 (2019): 1166–1179. 30808729 10.1158/1541-7786.MCR-18-1147

[71] S. Bai , S. Cao , L. Jin , et al., “A Positive Role of c‐Myc in Regulating Androgen Receptor and Its Splice Variants in Prostate Cancer,” Oncogene 38, no. 25 (2019): 4977–4989. 30820039 10.1038/s41388-019-0768-8 PMC6586509

[72] X. Li , J. B. Wu , Q. Li , K. Shigemura , L. W. K. Chung , and W. C. Huang , “SREBP‐2 Promotes Stem Cell‐Like Properties and Metastasis by Transcriptional Activation of c‐Myc in Prostate Cancer,” Oncotarget 7, no. 11 (2016): 12869–12884. 26883200 10.18632/oncotarget.7331 PMC4914327

[73] D. Westaby , M. L. D. Fenor de la Maza , A. Paschalis , et al., “A New Old Target: Androgen Receptor Signaling and Advanced Prostate Cancer,” Annual Review of Pharmacology and Toxicology 62 (2022): 131–153. 10.1146/annurev-pharmtox-052220-015912 34449248

[74] M. Hashemi , A. Taheriazam , P. Daneii , et al., “Targeting PI3K/Akt Signaling in Prostate Cancer Therapy,” Journal of Cell Communication and Signaling 17, no. 3 (2023): 423–443. 36367667 10.1007/s12079-022-00702-1 PMC10409967

[75] S. Sharan , K. Nikhil , and P. Roy , “Effects of Low Dose Treatment of Tributyltin on the Regulation of Estrogen Receptor Functions in MCF‐7 Cells,” Toxicology and Applied Pharmacology 269, no. 2 (2013): 176–186. 23523586 10.1016/j.taap.2013.03.009

[76] S. Singh , S. Sadacharan , S. Su , A. Belldegrun , S. Persad , and G. Singh , “Overexpression of Vimentin: Role in the Invasive Phenotype in an Androgen‐Independent Model of Prostate Cancer,” Cancer Research 63, no. 9 (2003): 2306–2311. 12727854

[77] S. Baritaki , A. Chapman , K. Yeung , D. A. Spandidos , M. Palladino , and B. Bonavida , “Inhibition of Epithelial to Mesenchymal Transition in Metastatic Prostate Cancer Cells by the Novel Proteasome Inhibitor, NPI‐0052: Pivotal Roles of Snail Repression and RKIP Induction,” Oncogene 28, no. 40 (2009): 3573–3585. 19633685 10.1038/onc.2009.214

[78] M. J. Bussemakers , A. van Bokhoven , K. Tomita , C. F. J. Jansen , and J. A. Schalken , “Complex Cadherin Expression in Human Prostate Cancer Cells,” International Journal of Cancer 85, no. 3 (2000): 446–450. 10652439

[79] C. G. Leon , J. A. Locke , H. H. Adomat , et al., “Alterations in Cholesterol Regulation Contribute to the Production of Intratumoral Androgens During Progression to Castration‐Resistant Prostate Cancer in a Mouse Xenograft Model,” Prostate 70, no. 4 (2010): 390–400. 19866465 10.1002/pros.21072

[80] J. H. Gunter , A. A. Lubik , I. McKenzie , M. Pollak , and C. C. Nelson , “The Interactions Between Insulin and Androgens in Progression to Castrate‐Resistant Prostate Cancer,” Advances in Urology 2012, no. 1 (2012): 248607. 22548055 10.1155/2012/248607 PMC3324133

[81] S. Wang , K. Li , Z. Liu , S. Gui , N. Liu , and X. Liu , “Aerobic Exercise Ameliorates Benign Prostatic Hyperplasia in Obese Mice Through Downregulating the AR/Androgen/PI3K/AKT Signaling Pathway,” Experimental Gerontology 143 (2021): 111152. 33189835 10.1016/j.exger.2020.111152

[82] Y. Furuya , Y. Sekine , H. Kato , Y. Miyazawa , H. Koike , and K. Suzuki , “Low‐Density Lipoprotein Receptors Play an Important Role in the Inhibition of Prostate Cancer Cell Proliferation by Statins,” Prostate International 4, no. 2 (2016): 56–60. 27358845 10.1016/j.prnil.2016.02.003 PMC4916060

[83] X. Wang , B. Sun , L. Wei , et al., “Cholesterol and Saturated Fatty Acids Synergistically Promote the Malignant Progression of Prostate Cancer,” Neoplasia 24, no. 2 (2022): 86–97. 34954451 10.1016/j.neo.2021.11.004 PMC8718564

[84] J. Swinnen , P. P. van Veldhoven , M. Esquenet , W. Heyns , and G. Verhoeven , “Androgens Markedly Stimulate the Accumulation of Neutral Lipids in the Human Prostatic Adenocarcinoma Cell Line LNCaP,” Endocrinology 137, no. 10 (1996): 4468–4474. 8828509 10.1210/endo.137.10.8828509

[85] M. W. O'Reilly , P. J. House , and J. W. Tomlinson , “Understanding Androgen Action in Adipose Tissue,” Journal of Steroid Biochemistry and Molecular Biology 143 (2014): 277–284. 24787657 10.1016/j.jsbmb.2014.04.008

[86] L. M. Butler , M. M. Centenera , and J. V. Swinnen , “Androgen Control of Lipid Metabolism in Prostate Cancer: Novel Insights and Future Applications,” Endocrine‐Related Cancer 23, no. 5 (2016): R219–R227. 27130044 10.1530/ERC-15-0556

[87] R. G. Uc‐Peraza , Í. B. Castro , and G. Fillmann , “An Absurd Scenario in 2021: Banned TBT‐Based Antifouling Products Still Available on the Market,” Science of the Total Environment 805 (2022): 150377. 34818813 10.1016/j.scitotenv.2021.150377

[88] P. Dowson , J. Bubb , and J. Lester , “Persistence and Degradation Pathways of Tributyltin in Freshwater and Estuarine Sediments,” Estuarine, Coastal and Shelf Science 42, no. 5 (1996): 551–562.

[89] W. Langston and N. Pope , “Determinants of TBT Adsorption and Desorption in Estuarine Sediments,” Marine Pollution Bulletin 31, no. 1–3 (1995): 32–43.

